# Lactate oxidative phosphorylation by annulus fibrosus cells: evidence for lactate-dependent metabolic symbiosis in intervertebral discs

**DOI:** 10.1186/s13075-021-02501-2

**Published:** 2021-05-21

**Authors:** Dong Wang, Robert Hartman, Chao Han, Chao-ming Zhou, Brandon Couch, Matias Malkamaki, Vera Roginskaya, Bennett Van Houten, Steven J. Mullett, Stacy G. Wendell, Michael J. Jurczak, James Kang, Joon Lee, Gwendolyn Sowa, Nam Vo

**Affiliations:** 1 Department of Orthopaedic Surgery, Ferguson Laboratory for Orthopedic and Spine Research, University of Pittsburgh, 200 Lothrop Street, E1644 Biomedical Science Tower, Pittsburgh, PA 15261 USA; 2 University of Pittsburgh Medical Center Enterprises, Pittsburgh, PA 15213 USA; 3 Tianjin Hospital, 406 Jiefang South Road Hexi District, Tianjin, PR China; 4 Department of Pharmacology and Chemical Biology, University of Pittsburgh, 5117 Centre Avenue, Pittsburgh, PA 15213 USA; 5 Health Sciences Metabolomics and Lipidomics Core, University of Pittsburgh, Pittsburgh, PA USA; 6 Department of Medicine, Division of Endocrinology and Metabolism, University of Pittsburgh, Pittsburgh, PA USA; 7 Center for Metabolism and Mitochondrial Medicine, University of Pittsburgh, Pittsburgh, PA 15213 USA; 8 Department of Orthopedics, Brigham and Womens Hospital, School of Medicine, Harvard University, 75 Francis Street, Boston, MA 02115 USA; 9 Department of Physical Medicine and Rehabilitation, University of Pittsburgh School of Medicine, Pittsburgh, PA 15219 USA

**Keywords:** Lactate, Lactic acid, Intervertebral disc, Annulus fibrosus, Nucleus pulpous, Glycolysis, Oxidative phosphorylation

## Abstract

**Background:**

Intervertebral disc degeneration contributes to low back pain. The avascular intervertebral disc consists of a central hypoxic nucleus pulpous (NP) surrounded by the more oxygenated annulus fibrosus (AF). Lactic acid, an abundant end-product of NP glycolysis, has long been viewed as a harmful waste that acidifies disc tissue and decreases cell viability and function. As lactic acid is readily converted into lactate in disc tissue, the objective of this study was to determine whether lactate could be used by AF cells as a carbon source rather than being removed from disc tissue as a waste byproduct.

**Methods:**

Import and conversion of lactate to tricarboxylic acid (TCA) cycle intermediates and amino acids in rabbit AF cells were measured by heavy-isotope (^13^C-lactate) tracing experiments using mass spectrometry. Levels of protein expression of lactate converting enzymes, lactate importer and exporter in NP and AF tissues were quantified by Western blots. Effects of lactate on proteoglycan (^35^S-sulfate) and collagen (^3^H-proline) matrix protein synthesis and oxidative phosphorylation (Seahorse XFe96 Extracellular Flux Analyzer) in AF cells were assessed.

**Results:**

Heavy-isotope tracing experiments revealed that AF cells imported and converted lactate into TCA cycle intermediates and amino acids using in vitro cell culture and in vivo models. Addition of exogenous lactate (4mM) in culture media induced expression of the lactate importer MCT1 and increased oxygen consumption rate by 50%, mitochondrial ATP-linked respiration by 30%, and collagen synthesis by 50% in AF cell cultures grown under physiologic oxygen (2-5% O_2_) and glucose concentration (1-5mM). AF tissue highly expresses MCT1, LDH-H, an enzyme that preferentially converts lactate to pyruvate, and PDH, an enzyme that converts pyruvate to acetyl-coA. In contrast, NP tissue highly expresses MCT4, a lactate exporter, and LDH-M, an enzyme that preferentially converts pyruvate to lactate.

**Conclusions:**

These findings support disc lactate-dependent metabolic symbiosis in which lactate produced by the hypoxic, glycolytic NP cells is utilized by the more oxygenated AF cells via oxidative phosphorylation for energy and matrix production, thus shifting the current research paradigm of viewing disc lactate as a waste product to considering it as an important biofuel. These scientifically impactful results suggest novel therapeutic targets in disc metabolism and degeneration.

**Supplementary Information:**

The online version contains supplementary material available at 10.1186/s13075-021-02501-2.

## Background

Intervertebral discs (IVDs) are fibrocartilaginous structures necessary for bearing loads and providing flexibility in the spine. Intervertebral disc degeneration (IDD) contributes to several major spine-related pathologies, including chronic low back pain, disability, and debilitating pain that have resulted in tremendous societal health and economic burden exceeding $100B annually in cost [1]. Among the possible drivers of IDD, impaired disc nutrition has long been investigated as a major contributor to the initiation and development of IDD [2].

The IVD is the largest avascular tissue structure comprised of a gelatinous nucleus pulposus (NP) center surrounded by an outer annulus fibrosus (AF). Disc cells receive their nutrients principally through passive diffusion from peripheral capillaries residing in the subchondral plates and outer AFmetabolic wastes are removed by the same mechanism [3]. NP and AF tissue have distinct metabolic and hypoxic environments due to their positions within the IVD. Being farthest from the capillary supply, the NP is hypoxic, while the AF is more oxygenated as it is located closer to the peripheral vasculature [2]. The NP contains the lowest glucose concentration and the highest lactate levels (510mM) because its cells metabolize glucose through anaerobic glycolysisto generate lactic acid which readilydissociatesto lactate andH+ due to its low pKa of 3.9. NP producesand secretes an abundant amount of lactate into the extracellular environment [4]. This results in lactate concentration being highest at the discs center and decreases in concentration going peripherally [5].

Accumulation of lactic acid acidifies the disc micro-environment, which can reduce disc cell viability and function. The pH range of non-degenerate disc tissue is typically 7.17.4. Increased acidity observed in degenerate disc tissue (pH6.26.8) has been reported to cause a decrease in cell proliferation and viability, leading to an increase in matrix catabolism and inflammation in human NP cells [6]. Increased acidity (pH<6.8) has been shown to dramatically suppress proteoglycan matrix synthesis in human and bovine NP tissue [7]. Acidic pH has recently been shown to promote cellular senescence in rat NP cell cultures [8], and senescent disc cells exhibit perturbed matrix homeostasis [9]. Hence, perturbation of disc matrix homeostasis by acidic pH could be mediated through cellular senescence. Based onthese observations, the longstanding dogma in the field presumesthat lactic acid is a toxic waste product of NP glycolysis that must be removed to maintain disc health [6, 7, 10, 11].

Thiswidespread presumption fails to consider several aspects of disc bioenergetics. First, lactate, is an energy rich metabolite, which can yield ~16 ATP per lactate molecule if converted to pyruvate and metabolized via oxidative phosphorylation (OXPHOS). Given the inherent nutrient-deprived disc environment, can disc cells afford to waste this energy resource? Second, how do disc cells survive and function in a high lactate milieu in vivo? Herein, we re-examined disc lactate metabolism in light of recent literature from solid tumor research that reported lactate metabolic symbiosis as an alternative metabolic strategy. Solid tumors share similar structural and metabolic features with the disc. The tumor center is hypoxic and glycolytic (structurally analogous to the NP), while the tumors outer layer is more oxygenated (analogous to the AF). Lactate metabolic symbiosis occurs in solid tumors whereby lactate produced by glycolytic cells from the hypoxic center is utilized through OXPHOS by cells in the more oxygenated outer part of the tumor [12,14]. Although reported relatively recently in solid tumors, the concept of lactate metabolic symbiosis has long been documented in muscle and brain physiology [15,17]. For instance, fast-twitch white muscle fibers have been shown to glycolytically convert glucose to lactate [18] that is then secreted and taken up to be aerobically metabolized by slow-twitch red muscle fibers [19]. Therefore, tissues living in different degrees of hypoxia have evolved to optimize their bioenergetics through lactate symbiosis.

In the present study, we postulate that IVDs employ lactate-dependent metabolic symbiosis as a metabolic adaptation to optimize their cellular bioenergetics, i.e. NP produces lactate via glycolysis and AF consumes lactate via OXPHOS as a biofuel to minimize lactate accumulation and its negative impact on NP cells. We found evidence of robust lactate uptake and OXPHOS by AF cells used to generate energy and amino acids. We also demonstrate that AF cells express the necessary molecular machinery for handling lactate, including monocarboxylate transporter 1 (MCT1) for lactate import and lactate dehydrogenase M (LDH-M) for converting lactate into pyruvate for OXPHOS. These new findings are significant in that they support a profound paradigm shift away from seeing lactate as waste in the disc to investigating its role as an alternative carbon source.

## Methods

### AF and NP cell cultures

Twenty-four 7- to 10-week-old female New Zealand White rabbits (*Covance*, Denver, PA, USA) were used for all experiments. Primary rabbit NP and AF cells were initially expanded on monolayer culture in Gibco Hams F12 media (11765047, *Thermo Fisher Scientific*) containing 25mM glucose and 10% FBS at 5% O_2_. Before being treated with different lactate experimental conditions, passage 1 (P1) cell cultures were conditioned in low glucose DMEM media (5.5mM glycose, D5921, *Sigma*) with 4mM exogenously added sodium lactate (S1324, *Spectrum Chemical MFG Corp*) to mimic disc physiological nutrient niche for 24h. Human AF (hAF) cells were isolated from disc surgical specimens (mean=42years, average Thompson degeneration grade 23) and cultured in the same manner as rabbit AF cells described above. P1 hAF cells were used to in the ^13^C-lactate tracing assay.

### Measurement of cell culture media glucose and lactate

P1 rabbit NP and AF cells were grown on monolayer cultures in Gibco Hams F12 containing 23mM glucose and 10% FBS at 5% O_2_. Glucose and lactate concentrations in the cell culture media were quantified at different time points (2, 6, 12, 24, 48, 72h) using Accutrend Plus System (*Roche Diagnostics*, Switzerland) for glucose (used 10l media/time point) and lactate (0.7l media/time point) [20].

### CCK8 cell viability assay to assess lactate tolerance by AF cells

P1 rabbit AF cells cultured in media containing 1.06mM glucose and 1% FBS were treated with different sodium-lactate concentrations (0, 2.5, 5, 7.5, 10, 20mM) for 48h at 5% O_2_. Cell viability was measured by CCK-8 assay (CK04, *Dojindo Molecular Technologies*, Inc., Rockville, MD, USA), following the manufacturers instructions. Cell morphology and density were also evaluated.

### ^14^C-lactate uptake assay

P1 rabbit AF cell culture in a 12-well plate was conditioned for 48h in low glucose (5.5mM glucose) DMEM media, 4mM lactate, and 10% FBS at 5% O_2_. AF cells were then pre-treated with an assay media containing 150mM NaCl, 5mM KCl, 1mM MgCl_2_, 1mM CaCl_2_, 25mM HEPES (pH7.4), and 0.5% FBS at 5% O_2_ for 30min. Cells were next labeled for 1 min in the absence of glucose with different lactate concentrations (1, 2, 4mM) at a constant ^14^C-lactate (NEC599050UC, *PerkinElmer*) specific activity (500Ci/mmole lactate). Glucose was left out at ^14^C-lactate labeling step because this assay was designed with the sole purpose of initially finding out whether AF cells are capable of importing lactate. The culture media was removed, the cell layer was washed three times with 0.5ml ice cold assay media, and 0.5ml of 0.1M NaOH was added, followed by 50l of 0.5M HCl to neutralize the solution before cell lysate was collected. 0.4ml of cell lysate was counted in the Tri-Carb 2100TR Liquid Scintillation Analyzer (*PerkinElmer*). The remaining cell lysate was used to calculate the protein concentration for normalization of cellular ^14^C-lactate uptake. Relative levels of lactate uptake by AF cells were quantitated using ^14^C radioactivity from the cell lysate normalized to total protein amount.

### ^13^C-lactate tracing assay

#### In vitro model

P1 AF cells were conditioned in low glucose (5.5mM) DMEM media with 1% FBS and 4mM lactate at 5% O_2_ for 24h before labeling. For heavy isotope labeling, AF cells were labeled 4mM 3-^13^C-lactate (CLM-1578-PK, *Cambridge Isotope Laboratories*) in media containing 1mM or 5mM glucose and 4mML-glutamine without pyruvate at 1% or 5% O_2_ for 24h. For controls, cells without heavy isotope labeling (1mM glucose, 4mM lactate) or labeled with U-^13^C-glucose (U, uniformly labeled. CLM-1396-PK, *Cambridge Isotope Laboratories*) in 1mM ^13^C-glucose and 4mM lactate) at 5% O_2_ for 24h were included. Metabolic quenching and extraction of polar metabolite were performed using ice cold 80% methanol in water with 0.1% formic acid at a ratio of 500L per 28.2cm^2^ surface area of cell monolayer.

#### Ex vivo model

To trace ^13^C in AF cells within their native extracellular matrix, ex vivo rabbit disc organs containing the superior vertebrae and inferior vertebrae were cultured in low glucose (5.5mM glucose) DMEM culture media to mimic in vivo circulatory glucose. The disc organs were either cultured in media containing 4mM ^13^C-lactate or injected with 5l of 40mM ^13^C-lactate into the NP region using the Hamilton syringe with a 25-gauge needle. The un-injected and injected disc organs were cultured for 3 days to allow permeation of ^13^C-lactate into the AF tissue. AF tissues were dissected and snap frozen before being analyzed by high resolution mass spectrometry (HRMS).

#### In vivo model

To trace ^13^C in AF cells in vivo, two caudal discs (C7-C8, C8-C9) in the tails of anesthetized Fischer 344 rats (*n*=3, 4-month-old male rats) were injected with 1l of 100mM ^13^C-lactate into the NP region using the airtight Hamilton syringe (#80266 Model 1702 LT Threaded Plunger SYR, *Hamilton*, MA, USA) and 25-gauge needle (#7750-16 Kel-F Hub Needle, *Hamilton*, MA, USA). The injection was estimated to give about 510mM ^13^C-lactate final concentration in the entire disc volume. The rats were sacrificed 3 days post-injection, and AF tissue from the injected and un-injected disc control (C6-C7, C9-C10) was harvested separately from NP tissue under a dissecting microscope and snap frozen. The frozen tissue was homogenized in ice cold 80% methanol in water with 0.1% formic acid at a ratio of 15L per mg, at 60hz for 1min in a FastPrep homogenizer with matrix A garnet and ceramic matrix (*MP Biomedical*, Irvine, CA) before being processed by HRMS as described below.

### Sample preparation for HRMS

D4-taurine, D3-alanine, D3-lactate, and D3-creatinine (*Sigma-Aldrich*) were added to the cell or tissue lysates as an internal standard for a final concentration of 100M. The supernatant fluid of the cell or tissue lysate was cleared of protein by centrifugation at 16,000*g*. Samples (2L) were subjected to online LC-HRMS analysis.

### LC-HRMS method

Samples were analyzed by untargeted LC-HRMS (University of Pittsburgh Health Sciences Metabolomics and Lipidomics Core). Samples were injected via a Thermo Vanquish UHPLC and separated over a reversed phase Thermo Hypercarb porous graphitic column (2.1100mm, 3.0m particle size) maintained at 50C. For the 20-min LC gradient, the mobile phase consisted of the following: solvent A (0.1% formic acid in water) and solvent B (0.1% formic acid in acetonitrile). The gradient was as follows: 012.0min 5% B, to 1000% B; 12.015.0min held at 100% B, 15.015.1100% to 5% B; 15.120.0min 5% B. Spectra was acquired on a Thermo IDX tribrid mass spectrometer, using both positive and negative ion mode, scanning in Full MS mode (2 scans) from 70 to 800*m/z* at 120,000 resolution with an AGC target of 5e4. Source ionization settings was 3.5/2.6kV spray voltage respectively for positive and negative mode. Source gas parameters was 20 sheath gas, 10 auxiliary gas at 300C, and 4 sweep gas. The calibration was performed prior to analysis using the Pierce Positive and Negative Ion Calibration Solutions (Thermo Fisher Scientific). Integrated peak areas were then extracted manually using Quan Browser (Thermo Fisher Xcalibur ver. 2.7). ^13^C enrichment and natural abundance corrections were calculated using previously established MIMOSA methodology [21]. Graphs and statistical analyses (either *t* test or ANOVA) were prepared with GraphPad Prism 7.0 (*GraphPad Software*, Inc., La Jolla, CA, USA). We measured enrichment or atoms percent excess (APE) for lactate, pyruvate, acetyl-CoA, citrate, succinate, and malate as well as the amino acids glutamate, glutamine, and valine.

### Western blots

AF and NP tissues were carefully isolated separately from lumbar discs of four 6-month-old female New Zealand White rabbits. Tissue protein extracts were prepared using T-PER Tissue Protein Extraction Reagent with proteinase inhibitor cocktail as per the manufacturers instructions (Cat. No 78510, *Thermo Fisher*). Western blots were performed as described previously [22] to detect hexokinase-1 (Anti-HK, Ab150423, *Abcam*), MCT4 (anti-MCT4, SC-376140, *Santa Cruz Biotechnolgy*), LDHA (anti-LDHA, PA5-27406, *Invitrogen*), MCT1 (anti-MCT1, AB93048, *Abcam*), LDHB (anti-LDHB, Ab85319, *Abcam*), and pyruvate dehydrogenase (PDH; anti-PDH, #2784S, *Cell Signaling Technology*). Loading control -actin (Cat. No. PA1-183, *Thermo Fisher*) and anti-rabbit HRP secondary antibody (Cat. No. 31460, *Thermo Fisher*) were used.

### Bioenergetic flux measurement by Seahorse XFe96

Four independent P1 AF cells from four rabbits were cultured in low-glucose DMEM containing 5.5-mM glucose and 10% FBS at 5% O_2_ in 4mM lactate. AF cell bioenergetic flux in the presence and absence of lactate was measured by using Seahorse XFe96 Analyzer as previously described [23]. Oxygen consumption rate (OCR) was calculated and normalized to protein amount as measured using the Crystal Violet dye (C3886, *Sigma-Aldrich*). Individual parameters of OXPHOS were derived from OCR profiles, including mitochondrial respiration-mediated ATP production, as described [23, 24].

### Matrix protein synthesis by AF cells

Four independent P1 AF cells from four rabbits were cultured in 4mM lactate in 1 or 5mM glucose at 5% O_2_ for 3 days in 0.5ml of F-12/DMEM containing 10% FBS, 1% PS, and 25g/mlL-ascorbic acid in a 48-well plate in the presence of 20Ci/ml ^35^S-sulfate (to measure proteoglycan synthesis) and 10Ci/ml collagenase-sensitive ^3^H-proline (to measure collagen synthesis) at 37C. Assays of proteoglycan and collagen syntheses by AF cells were performed as previously described [25]. The rate of synthesis was calculated as fmoles of proline (collagen synthesis) or sulfate (proteoglycan synthesis) incorporated per g DNA as measured using the QuantiT PicoGreen dsDNA Assay Kit (P7589, *Life Technologies*).

## Results

### Rationale

This study is motivated by our new hypothesis postulating that lactate produced by NP glycolysis is not a waste end-product in the IVD but rather is an important and precious carbon source for OXPHOS in AF cells residing in a nutrient-poorbut more oxygenated disc environment. We also hypothesize that utilization of lactate by AF cells also serves to minimize NP lactate accumulation and its negative impact on NP cells. To investigate our postulated lactate-dependent metabolic symbiosis between NP and AF, we examined the capacity of AF cells to uptake and utilize lactate using in vitro AF cell and ex vivo disc organ culture model systems. Both radioactive and stable isotope tracing by HRMS were employed for this purpose. Rabbit models were used as they provide enough disc cells for cell cultures, and their disc size is sufficiently large for the ^13^C-lactate injection and tracing study. Additionally, we decided to perform initial characterization of lactate metabolic symbiosis in young normal discs, which are readily available in rabbits but not in humans.

### AF cells are less glycolytic than NP cells

**Fig. 1:**
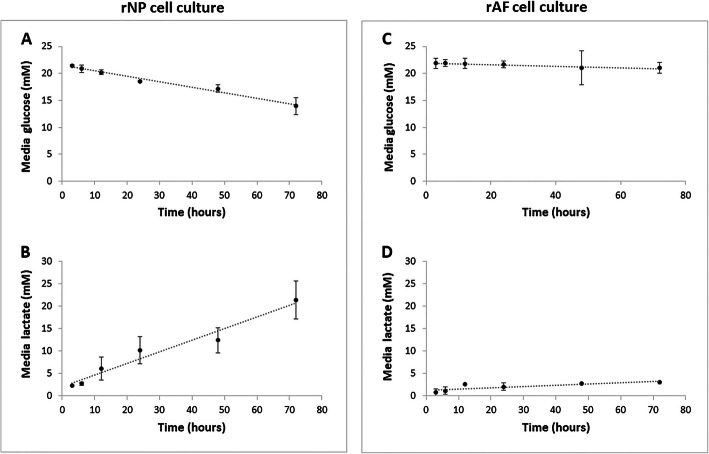
Distinct metabolic features of NP and AF cells in cell culture. Rabbit NP and AF cells were grown on monolayer cultures in Gibco Hams F12 media at 5% O_2_, and the concentrations of glucose and lactate in their culture media were measured by Accutrend Strips at different time points. NP cells in cell culture are highly glycolytic that readily consumed glucose (**a**) and produced and secreted an abundance of amount of lactate (**b**) in a time-dependent manner. Under the same culture condition, AF cells both consumed glucose (**c**) and produced lactate (**d**) at much slower rates compared to NP cells, suggesting that AF cells are less glycolytic and more aerobic in their metabolism

Because we used rabbit nucleus pulposus (rNP) and annulus fibrosus (rAF) cell cultures as the model system in our study, we first needed to confirm that these cell types in our in vitro model exhibit metabolic features consistent with those previously reported [23, 26]. Indeed, rNP cells grown in culture at 5% O_2_ readily consumed glucose (Fig.1a) and then produced and secreted lactate at a high steady-state rate (Fig. 1b). In contrast, AF cells grown under the same condition produced a much lower amount of lactate (Fig. 1d), but also consumed glucose at a much slower rate than NP cells (Fig. 1c) even though AF cells proliferate at similar or faster rates than NP cells under these conditions [23, 27, 28]. These results suggest that AF cells are less glycolytic than NP cells and that AF cells use less glucose than NP cells, likely because AF cells utilize OXHPOS which generates more ATP per glucose than glycolysis.

### AF cells tolerate high lactate levels

In anaerobic NP tissue, pyruvate generated from glycolysis is converted to lactic acid. Given its low pKa of 3.9, lactic acid readily dissociates into lactate and H+ which acidifies the disc tissue. It is the acidity created by lactic acid, not lactate, that is reported to cause harm to cells. Physiological lactate concentrations in human disc tissue from periphery to the center have been reported to range from 2 to 16mM while glucose concentrations range from 1 to 5mM [4]. To determine if rAF cells in culture could tolerate lactate in this physiologic range, rAF cell cultures were exposed to media containing exogenously added lactate ranging from 0 to 20mM lactate in the presence of physiologic glucose concentration (1.06mM). Lactate up to 10mM had no effects on AF cell viability in culture as assessed by CCK8 assay (Figure S1. B, Supplementary Material). Only at high lactate (20mM) concentration did we observed a modest decrease in AF cell viability by about 20%. Lactate up to 10mM also had no discernable effects on AF cell morphology or density in vitro (Figure S1. A, Supplementary Material). Given that normal blood lactate concentration is 0.51mM, these results suggest that AF cells have evolved to tolerate high lactate concentrations in disc tissue that are 510 times the level found in serum [5]. Because AF physiologic lactate concentrations range mostly between 2 and 6mM [4], we chose 4mM lactate to test its effects on AF cell metabolism in all our subsequent experiments.

### Lactate uptake by AF cells

**Fig. 2:**
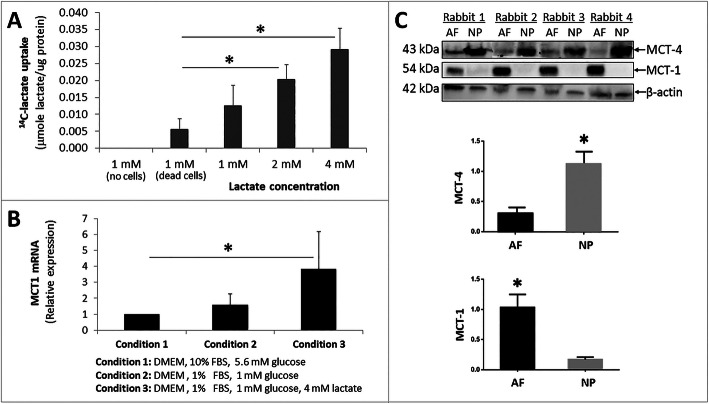
Lactate import into AF cells. **a**, ^14^C-lactate radioactive tracing to assess lactate import into AF cells. Rabbit AF cell cultures were grown in 1mM glucose and varying concentrations of ^14^C-lactate (14mM) for 1 min, washed with PBS, lysed, and counted in scintillation fluid. **b**, Lactate enhances MCT1 mRNA expression in AF cells. Rabbit AF cells were cultured under three different conditions, and their MCT1 mRNA levels were measured by qRT-PCR. **c**, Western blot of MCT-1 and MCT-4 in rabbit AF and NP tissue protein extract (top) and quantification of MCT-1 and MCT-4 expression levels by normalizing to -actin as loading control (bottom). Data are means SEM of four independent experiments (4 rabbits). * *p*<0.05

To determine whether AF cells can import lactatefrom their extracellular environment, we performed a radioactive tracing assay using ^14^C-lactate to measure cellular ^14^C uptake. Rabbit AF cells exposed to increasing ^14^C-lactate concentrations resulted in a proportional increase in ^14^C levels in the cells after the cells were extensively washed with PBS to remove nonspecific binding of ^14^C lactate (Fig.2a). To be sure that uptake was not due to nonspecific attachment of ^14^C-lactate to cells or plastic surface of the culture plate, we also included no-cell and dead cell controls, e.g. AF cells killed with 40% ethanol. These control samples showed minimal radioactive counts (Fig. 2a), suggesting negligible nonspecific binding of ^14^C lactate to AF cells. HepG2 cells, a human hepatocyte carcinoma cell line known to import lactate, were also included as a positive control for our uptake assay which resulted in a ^14^C-lactate-concentration dependent increase in radioactive counts in the cell lysate, as expected (Figure S2, Supplementary Material). The results from our ^14^C-lactate uptake assay demonstrated lactate import into AF cells.

Lactate import into cells is mediated preferentially via the monocarboxylate transporter 1 (MCT1) [29, 30]. To determine if AF cells express MCT1, we performed qRT-PCR and found higher MCT1 mRNA expression in AF cells cultured in the presence of lactate (Fig. 2b). Additionally, AF but not NP tissue expresses an abundant amount of MCT1 protein (Fig. 2c). Our findings suggest that AF has the molecular machinery and capability to import lactate from the extracellular environment. In contrast, MCT4 protein, a known lactate exporter in hypoxic tissue [12, 31, 32], is expressed mostly in NP but not AF tissue (Fig. 2c). These data support the notion of lactate metabolic synergy between NP and AF tissues whereby NP produces and exports lactate via MCT4 into the extracellular space, which is then imported into AF cells via MCT1.

### Lactate conversion to pyruvate by AF cells

**Fig. 3:**
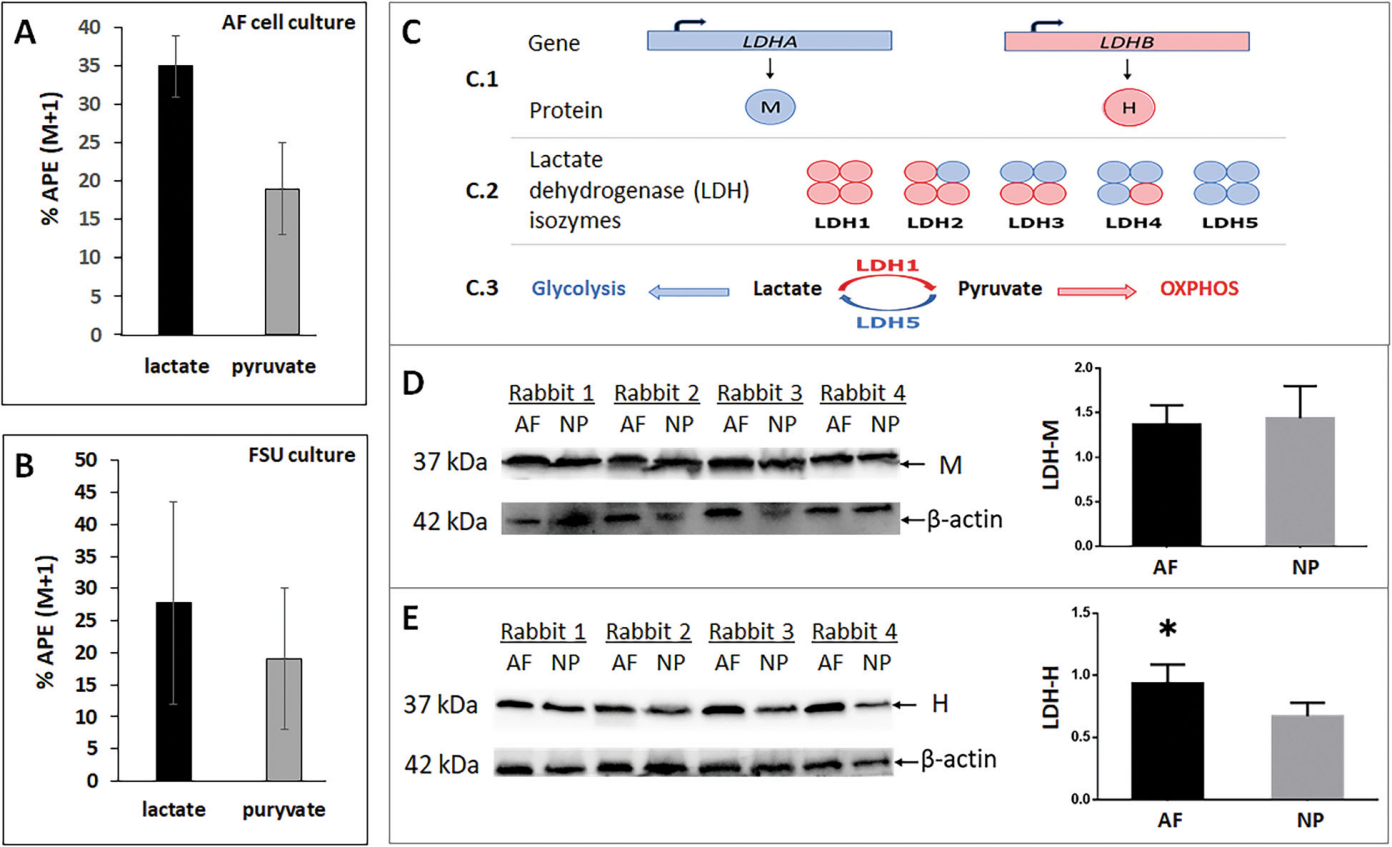
Lactate conversion to pyruvate in AF cells. ^13^C-lactate tracing to pyruvate in rabbit AF cells in cell culture containing 4mM 3-^13^C-lactate (**a**) and in AF tissue extract derived from the rabbit functional spine unit (FSU) culture 3days post injection of 5l of 40mM ^13^C-lactate into the NP region of the disc (**b**). Intracellular enrichment of ^13^C lactate or ^13^C pyruvate AF cells from AF cell culture or AF tissue of the ex vivo disc organ culture is reported as atomic percent excess (APE) of the total amount of lactate or pyruvate, e.g., 10% APE of pyruvate indicates 10% of total pyruvate contains ^13^C. Percent (%) APE shown (M+1) indicates that one ^13^C carbon is present. **c**, Schematic of gene expression and assembly of lactate dehydrogenase (LDH), the primary enzyme that catalyzes the interconversion of lactate and pyruvate. LDH exists in five isozymes composed of a tetramer of M and H protein subunits encoded by the LDHA and LDHB genes, respectively. LDH5, composed of four M subunits, preferentially converts pyruvate to lactate while LDH1, consisting of four H subunits, preferentially converts lactate to pyruvate. Western blot of LDHM (**d**) and LDHH (**e**) in rabbit AF and NP tissue protein extract and their protein levels were quantified by normalizing to -actin as loading control (graphs). Data are means SD of four independent experiments (four rabbits). * *p*<0.05

Because AF cells reside within a more oxygenated region of the disc, we postulate that AF cells convert lactate back into pyruvate for its subsequent conversion to acetyl-coA to be shuttled in the TCA cycle for OXPHOS. To test this idea, we performed stable isotope tracing using 3-^13^C-lactate by HRMS. AF cell cultures labeled with 4mM 3-^13^C-lactate for 24h resulted in 354% atomic percent enrichment (APE) of M+1 lactate and 196% APE of M+1 pyruvate (Fig.3a). M+1 indicates that one ^13^C carbon is present in these molecules. These findings confirmed that lactate is taken up and converted into pyruvate by AF cells in an in vitro cell culture model system.

To further demonstrate that AF cells in their native tissue are also capable of importing lactate and converting it to pyruvate, we performed stable isotope ^13^C-lactate labeling using an ex vivo disc organ culture model. Rabbit functional spine units (FSUs) containing vertebrae-disc-vertebrae were injected with 3-^13^C-lactate into the NP region to give an estimated final 3-^13^C-lactate concentration of ~510mM. The FSUs were then incubated in the culture media for 3 days before being analyzed by HRMS. Under these conditions, there was a 2815% enrichment in M+1 ^13^C-lactate and 199% APE in M+1 pyruvate in AF tissue (Fig. 3b). As a control, we also incubated rabbit FSUs in culture media containing 4mM 3-^13^C-lactate for 3 days which resulted in 689% enrichment in M+1 ^13^C-lactate and 487% APE in M+1 pyruvate in AF tissue extract (Figure S3, Supplementary Material). These findings demonstrated that AF cells in their native tissue environment can uptake and convert lactate into pyruvate.

Lactate dehydrogenase isozyme 1 (LDH1), a homo-tetramer of four H protein subunits, preferentially converts lactate to pyruvate (Fig. 3c) [33, 34]. H is expressed significantly more in AF than NP tissue as shown by Western blot analysis (Fig. 3e), which is consistent with ^13^C being traced to pyruvate in our ^13^C-lactate tracing experiment. However, the presence of H protein in NP tissue suggests that NP cells also are enzymatically capable of converting lactate to pyruvate to a certain extent. LDH5, an isozyme consisting of a homo-tetramer of four M protein subunits, preferentially converts pyruvate to lactate [33, 34]. We expected the M protein to be expressed mostly in the hypoxic NP and less so in AF tissue. Surprisingly, this was not the case as M is expressed similarly in both NP and AF tissue (Fig. 3d), suggesting that AF cells possess as as much enzymatic capability as NP cells to convert pyruvate to lactate.

**Fig. 4:**
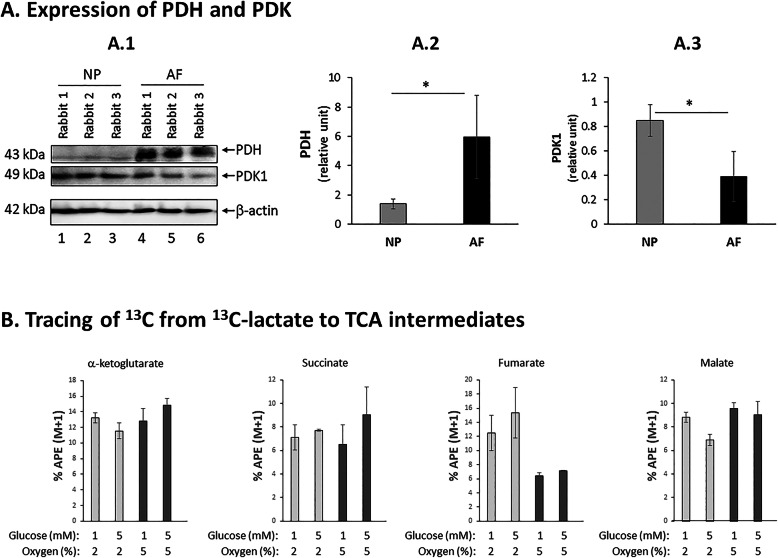
Lactate uptake and conversion to TCA intermediates and amino acids in AF cells. **A** PDH and PDK1 protein expression in AF and NP tissues. Western blot of PDH and PDK1 in rabbit AF and NP tissue protein extract (**A.1**), and PDH (**A.2**) and PDK1 (**A.3**) protein levels were quantified by normalizing to -actin as loading control (graphs). Data are means SD of three independent experiments (three rabbits). * *p*<0.05. **B** ^13^C from ^13^C-Lactate was traced to TCA intermediates and amino acids in AF cells. Rabbit AF cell cultures grown in 2% or 5% O_2_ were labeled with the stable isotope 3-^13^C-lactate (4mM) in the presence of 1mM or 5mM glucose; ^13^C was traced to different TCA intermediates by HRMS. Intracellular enrichment of ^13^C on different metabolites in AF cells was measured in APE or atom percent excess. Data are means SEM of four independent experiments (four rabbits)

Although pyruvate can enter the triccyclic acidic (TCA) cycle through its conversion to oxoaloacetate by pyruvate carboxylase, pyruvate primarily enters the TCA cycle through its conversion to acetyl-coA (Fig.6). The enzyme responsible for catalyzing the conversion of pyruvate to acetyl-coA is pyruvate dehydrogenase (PDH). As expected, PDH is expressed three fold more in AF than NP tissue (Fig.4A). Conversely, pyruvate dehydrogenase kinase 1 (PDK1), an enzyme that phosphorylates and inhibits PDH activity, is expressed about twofold more in NP than AF tissue (Fig.4A). Together, these results are consistent in indicating that AF cells, much more so than NP cells in disc tissue, possess the molecular machinery necessary for importing lactate through MCT1, converting lactate to pyruvate by LDH1, and converting pyruvate to acetyl-coA by PDH.

### Lactate conversion to TCA intermediates and amino acids by AF cells

Our ^13^C-lactate tracing experiment using a rabbit AF cell culture model also revealed that ^13^C was present in several tricarboxylic acid (TCA) intermediates, including -ketoglutarate, succinate, fumarate, and malate at 615% APE range (Fig. 4B). In addition, intracellular enrichment of ^13^C on these four TCA intermediates in AF cells was negligibly affected by 1mM vs. 5mM glucose or 2% vs. 5% oxygen. Detection of ^13^C label in these TCA metabolites, with -ketoglutarate, succinate and fumarate being the precursors of malate, might be due to the unfavorable thermodynamic reaction of converting malate to oxaloacetate by malate dehydrogenase (G=+6.7kcalmol) [35], resulting in the buildup of these TCA intermediates (Fig. 6). The presence of lactate-handling enzymes combined with the observed production of TCA cycle intermediates from lactate in AF cells provide strong support that these cells are capable of importing and aerobically metabolizing lactate as a carbon source.

In addition to detecting heavy isotope labeling in the TCA intermediates, ^13^C was also traced to several amino acids in AF cell culture labeled with ^13^C-lactate. These include M+1 glutamate (315% APE), M+1 glutamine (0.50.2% APE), and M+1 alanine (4.20.2 APE) (Figure S4A, Supplementary Material). These results suggest that AF cells can utilize lactate to make amino acids since alanine biosynthesis can be derived from pyruvate and both glutamine and glutamate can be enzymatically derived from -ketoglutarate [36] (Fig. 6).

Using the same ^13^C-lactate tracing experiment, we also traced ^13^C to the malate and glutamate in human AF cell culture (Figure S4C, Supplementary Material), and more importantly in rat AF tissues in vivo (Figure S4D, Supplementary Material) to approximately 10% APE. Together, these findings demonstrated that AF cells, in both in vitro cell culture and in vivo models, can uptake and convert lactate to TCA intermediates and amino acids, and that this metabolic phenotype appears to be universal, i.e. not species specific.

### Lactate oxidative phosphorylation by AF cells

**Fig. 5:**
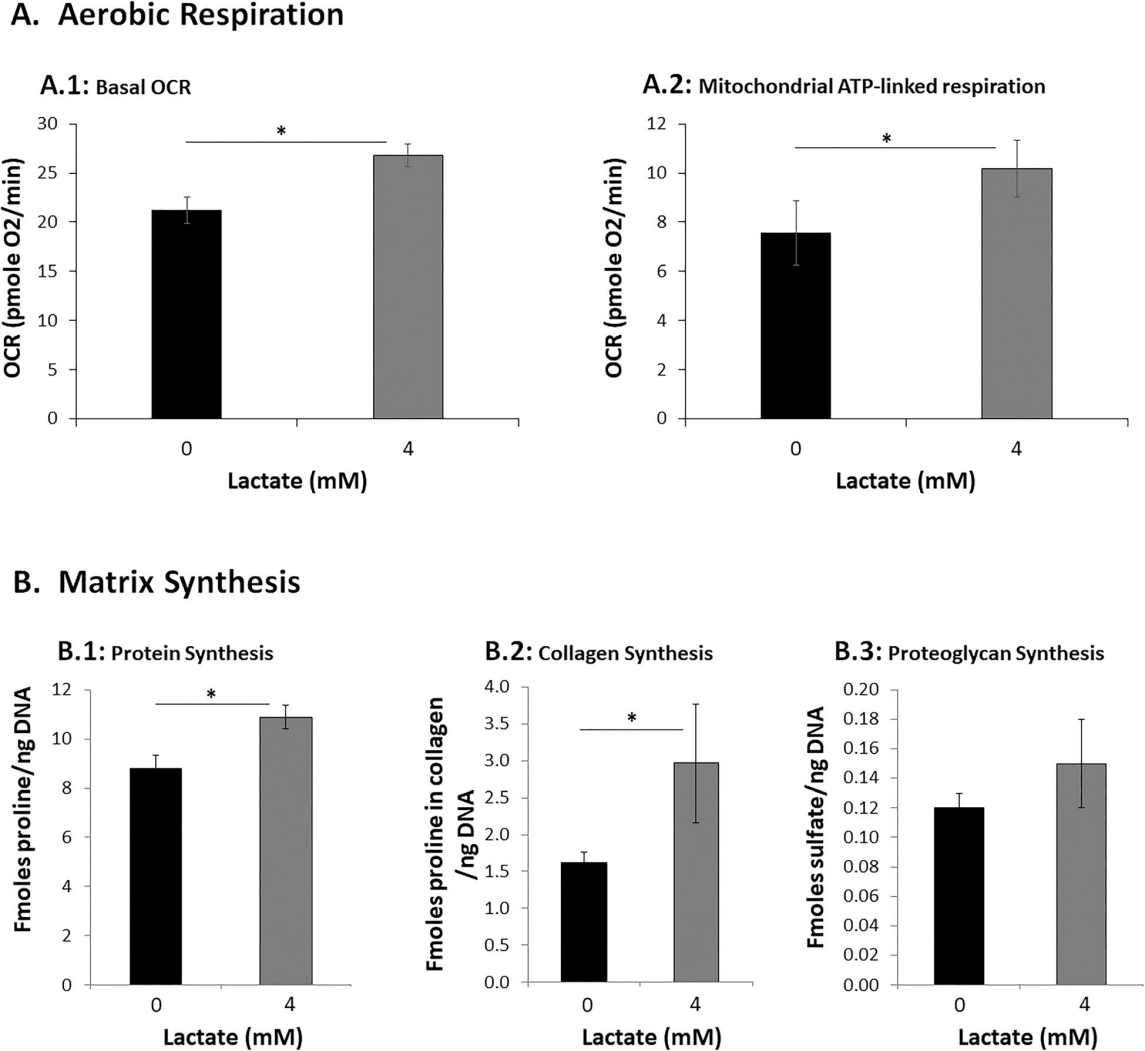
Lactate-treated AF cells exhibit increased aerobic respiration and matrix protein synthesis. **A** Increase in in basal oxygen consumption and mitochondria ATP-link respiration in AF cells treated with lactate. Oxygen consumption rates (OCR) of AF cells were measured by Seahorse XFe96 Extracellular Flux Analyzer at basal conditions and with serial administration of 1M oligomycin, 0.3M FCCP, 100mM 2DG, and 1M rotenone. OCR was calculated and normalized to protein amount and the results were expressed as a mean of four different samples SEM. Individual parameters of OXPHOS were derived from OCR profiles of AF cells lactate, as described in the Materials and methods section. Addition of lactate significantly increased basal OCR (**A.1**) and mitochondrial ATP-linked respiration (**A.2**). Results are expressed as mean of four different samples (derived from four rabbits)SEM, *p<0.05. **B** Exogenously added lactate increases matrix protein synthesis. Rabbit AF cell cultures exposed to physiological concentration of glucose (1mM) in the presence or absence of 4mM lactate. The presence of lactate also increases protein synthesis (^3^H-L-proline incorporation (**B.1**), collagen matrix synthesis (^3^H-Lproline incorporation & collagenase sensitive fraction (**B.2**), and proteoglycan synthesis (^35^S-sulfate incorporation (**B.3**). Results are expressed as mean of four different samples (derived from four rabbits)SEM. *p<0.05

Conversion of lactate into TCA intermediates by AF cells implies oxidative phosphorylation (OXPHOS) of lactate. However, ^13^C from ^13^C-lactate was also traced to amino acids, suggesting that lactate is used by AF cells for biosynthesis in addition to being used in OXPHOS to generate ATPs. To determine directly if lactate is used for OXPHOS, rabbit AF cells cultured in 1mM glucose 4mM lactate were analyzed using the Seahorse XFe96 Extracellular Flux Analyzer. Oxygen consumption rate (OCR), which reflects the extent of OXPHOS, was measured at basal conditions and following addition of specific inhibitors of the electron transport chain (Figure S5A, Supplementary Material) [37]. Under these conditions, lactate increased the basal OCR rate and the mitochondrial ATP-linked respiration in AF cells (Fig.5A), but it did not have any significant effects on several individual parameters of OXPHOS, including reserve capacity, maximum total respiratory capacity, proton leak, non-glucose respiration, and nonmitochondrial oxygen consumption (Figure S5B, Supplementary Material). These findings provide further evidence of lactate metabolism via OXPHOS to generate ATP in AF cells.

### Lactate increases matrix synthesis in AF cells

Lactate increases OXPHOS and mitochondrial ATP-linked respiration, as well as production of amino acids in AF cells when they were grown under the physiological nutrient condition of low glucose (1mM). A vital function of AF cells is to synthesize extracellular matrix, particularly the collagens, a process requires energy and amino acid building blocks. These observations raised a question of whether lactate can serve as a biofuel for matrix synthesis in AF cells. Indeed, our matrix synthesis assays using radioactive tracers revealed that AF cells synthesized almost twice as much as the total collagen (Fig. 5B, panel B.2) in the presence of 4mM lactate than without when cells were grown in low glucose (1mM) that mimic disc nutrient niche. Likewise, lactate stimulated total protein synthesis in AF cells to about 20% under the same condition (Fig. 5B, panel B.1). Interestingly, proteoglycan synthesis was also slightly increased in the presence of lactate but was not statistically significant (Fig. 5B, panel B.3). In addition, AF cells cultured in 5mM lactate produced collagen and total proteins to a comparable level to that found in AF cells cultured in 5mM glucose; and AF cells cultured in 1mM glucose and 4mM lactate, which add up to a total of 5mM between these two carbon sources, exhibited a slight but statistically nonsignificant decrease in collagen and total protein synthesis compared to AF cells grown in 5mM lactate or 5mM glucose (Figure S6D, Supplementary Material). Together, these findings demonstrate that under physiologic glucose concentration AF cells utilize lactate nearly as efficiently as glucose as a biofuel to produce matrix protein. In contrast, lactate treatment of NP cell cultures decreased overall matrix synthesis in these cells, albeit statistically nonsignificant, suggesting that NP cells do not metabolize lactate to make matrix (Figure S6, panels A-C in Supplementary Material).

## Discussion

Hypoxic NP cells produce and secrete large quantities of lactic acid into the extracellular environment of IVDs. Lactic acid acidifies tissue and NP cells have recently been shown in mice to adapt to acidic pH through the action of the proton/lactate exchange pump MCT4 to maintain intracellular pH [38]. The mechanism by which AF cells adapt in order to function in high lactic acid milieu and the associated acidity in disc tissue is largely unknown [39]. Until now, it has been commonly believed that disc tissue excretes lactate as an end waste product to prevent cytotoxic buildup. Here, we provided clear evidence through in vitro and in vivo experiments demonstrating that lactate can be imported into AF cells and metabolized via OXPHOS to generate ATPs and amino acids. We also demonstrated that lactate stimulates collagen and protein synthesis in AF cells cultured under limited nutrient, e.g. 1mM glucose and 1% FBS, that mimics the physiological condition. Thus, while lactate is an end-product of NP glycolysis, it represents an important biofuel for disc AF cells. Moreover, lactate utilization by AF cells can mitigate the buildup of lactate and its negative effects on NP cells, thus providing symbiotic benefits between AF and NP tissues. It should be noted that glycogen is also present in disc tissue, but it is primarily found within the NP tissue and minimally in the inner AF region [40]. Thus, glycogen most likely serves as a biofuel reserve for NP but not AF cells.

### Lactate import into AF cells

Through radioactive and heavy isotope lactate labeling and tracing experiments using in vitro cell culture and in vivo rat models, we demonstrated that AF cells are capable of importing lactate from the extracellular environment (Figs.2, 3, and 4, S4). Moreover, through immunoanalyses, we demonstrated that AF cells in their native tissue environment express the proper transporter required for importing lactate; rabbit AF but not NP tissue, expresses an abundance of MCT1 (Fig. 2). MCT1 serves the major physiological role in facilitating L-lactate transport into or out of cells depending on their tissue niche and metabolic state. However, this bidirectionality of MCT1 is reported to shift toward primarily lactate import in oxygenated tissues with mitochondrial OXPHOS capacity, including the peripheral region of solid tumors [12, 41], heart tissue [42], red skeletal muscle [43], liver parenchymal cells, and kidney convoluted tubule cells [29]. Given the fact that AF tissue is more oxygenated because it is closer to the vasculature and that AF cells harbor active mitochondria and carry out OXPHOS [23, 44, 45], our findings strongly suggest that MCT1 is the key lactate importer in AF cells. It should be noted that MCT2 and MCT3 are the other major lactate transporters with high lactate affinity with Km values lower or comparable to that of MCT1 [46]. However, expression of MCT2 and MCT3 was not detectable in disc tissue (data not shown). MCT4 is another lactate transporter, but it is hypoxia-inducible and predominates over MCT1 for lactate export in hypoxic and glycolytic tissues [38].

### Lactate metabolism in AF cells

Through ^13^C-lactate tracing experiments in vitro and in vivo, we demonstrated that AF cells can import and aerobically metabolize lactate (Figs.3 and 4). Interestingly, AF cells appear to use lactate mainly for biosynthesis and OXPHOS for energy production. This is evident by our detection of ^13^C in amino acids and TCA intermediates (Fig. 4B, Figure S4A). Interestingly, intracellular enrichment of ^13^C on different TCA metabolites from ^13^C-lactate labeling was largely unaffected when AF cells were cultured in 1mM or 5mM glucose or in 2% versus 5% oxygen. These findings suggest uptake and aerobic metabolism of lactate by AF cells is not significantly impacted by fluctuation of glucose and oxygen as long as such fluctuation stays within disc AF tissue physiologic range for glucose (15mM) and oxygen (25%). Moreover, our data also demonstrate that under disc physiologic glucose concentration (15mM), AF cells have similar preferences for utilizing lactate and glucose for oxidative phosphorylation and matrix synthesis (Figures S4B, S6D).

Our immunoanalyses also demonstrated that AF cells in their native tissue express the proper enzymes required for converting lactate back to pyruvate and to acetyl-CoA for OXPHOS (Figs. 2, 3, and 4). Lactate dehydrogenase (LDH), the primary enzyme that catalyzes the interconversion of lactate and pyruvate, exists in five isozymes composed of a tetramer of M and H protein subunits encoded by the LDHA and LDHB genes, respectively (Fig. 3c). Compared to NP, AF tissue expresses significantly more M protein that constitutes the LDH1 isozyme that preferentially converts lactate to pyruvate [34]. AF tissue, not NP, also highly expresses pyruvate dehydrogenase (PDH), the enzyme responsible for catalyzing the conversion of pyruvate to acetyl-coA for oxidative metabolism in the TCA cycle. On the other hand, AF but not NP tissue expresses low level of pyruvate dehydrogenase kinase 1 (PDK1) whose function is to inhibit PDH activity. Together, our findings support the idea that AF cells, much more so than NP cells in disc tissue, are programed to express the proper molecular machinery to import and convert lactate to acetyl-coA necessary for OXPHOS.

### A new model of disc metabolism: lactate-dependent metabolic symbiosis

**Fig. 6:**
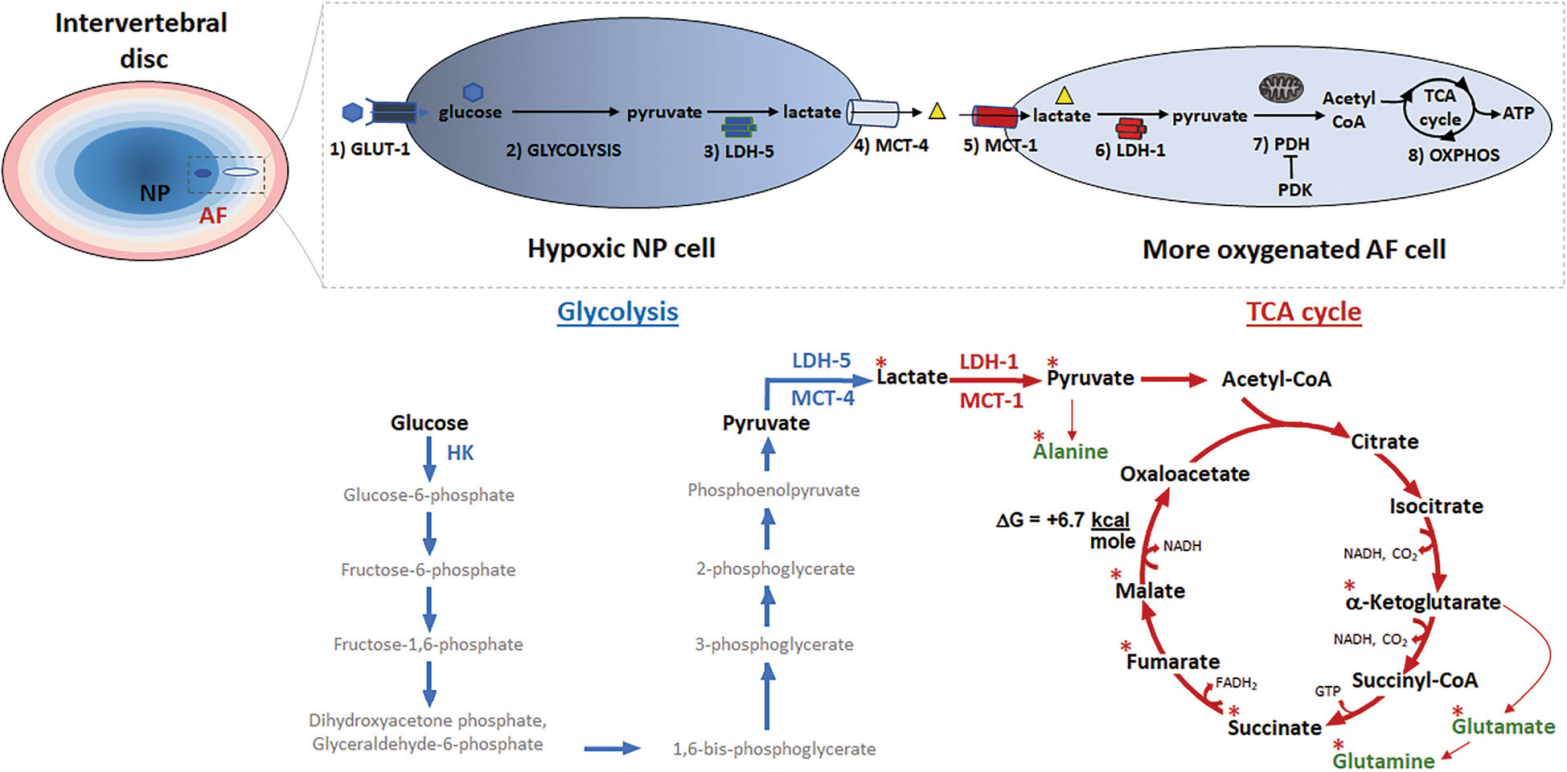
Proposed model of disc lactate metabolic symbiosis. A simplified model of disc lactate-dependent metabolic symbiosis showing the major steps of glucose metabolism starting with (1) glucose uptake via the Glut1 transporter into hypoxic NP cells, (2) conversion of glucose to pyruvate by glycolysis, (3) conversion of pyruvate to lactate by LDH5, (4) export of lactate out of NP cells through MCT4, (5) import of extracellular lactate into the more oxygenated AF cells via the MCT1, (6) conversion of lactate into pyruvate by LDH1, (7) conversion of pyruvate into acetyl-coA by PDH, which is negatively regulated by PDK, and (8) shuttle of acetyl-coA into the TCA cycle to generate precursors for biosynthesis and/or for oxidative phosphorylation to generate ATP. Below NP cell is shown the glycolytic pathway while below AF cell is the TCA cycle. Pyruvate to lactate converting enzyme (LDH-5), lactate exporter (MCT-4), lactate importer (MCT-1), and lactate to pyruvate converting enzyme (LDH-1) are shown. *Denotes the amino acids (green) and TCA metabolites (black) containing ^13^C derived from the heavy isotope ^13^C-lactate label. Malate conversion to oxaloacetate is the most thermodynamically unfavorable step in the TCA cycle with the G=+6.7kcalmol

Based on our new findings, we propose a working model of disc lactate-dependent metabolic symbiosis whereby hypoxic NP cells anaerobically convert glucose into lactate, which is then secreted and imported into neighboring cells of the more oxygenated AF tissue to be metabolized via OXPHOS (Fig. 6). For the sake of simplicity, this model illustrates the distinct metabolic features between NP and AF and highlights key transporters and enzymes most relevant for disc lactate-dependent metabolic symbiosis, although we acknowledge that such simple metabolic distinction might not be representative of what actually occurs in vivo given the gradients of oxygen and nutrient concentrations that exist in disc tissue. In hypoxic NP cells, glucose is taken up via the glucose transporter Glut1 [47] and converted to pyruvate through glycolysis. Pyruvate is then converted to lactate and exported into the extracellular space via MCT4. Each of these key steps is well documented by our data and previously published literature [4, 38, 47]. Glut1 is highly expressed in NP tissue [48], as hexokinase 2 (Figure S7, Supplementary Material), a key glycolytic enzyme, confirming the glycolytic nature of NP cells [38, 45]. Lactate export is likely mediated via MCT4 in NP cells as NP but not AF tissue highly expresses MCT4 (Fig. 3). Indeed, using transgenic MCT4 null mice, Silagi et al. recently demonstrated the important role of MCT4 as a lactate/proton co-exporter to maintain intracellular pH homeostasis in NP tissue [38]. Our proposed role of MCT4 as a lactate exporter in the hypoxic NP is also consistent with the literature of muscle and solid tumors, which reported that MCT4 (K_m_ lactate ~25mM) is hypoxia-inducible and predominates over MCT1 (K_m_ lactate ~5mM) for lactate export in glycolytic tissues [34, 49]. Expression of these proteins, MCT4, Glut1, and hexokinase, are upregulated by the hypoxia-inducible factor 1 (HIF-1) that is constitutively active in NP [50]. Altogether, these findings demonstrate the glycolytic phenotype of NP in producing and exporting lactate.

Our study has several limitations. First, a caveat to our approach is that by relying on isotopic enrichments alone, we are limited to calculations of relative flux. As recently described, relative flux calculations can overestimate net flux due to isotopic exchange [51]. Future studies to address the net contribution of lactate to amino acid synthesis and OXPHOS will be necessary. Second, lactate flux does not occur only in the AF and NP interface; in large discs such as human lumbar discs, lactate exit and glucose entrance also occur substantially via the endplate. Nevertheless, efficient utilization of lactate by AF tissue would ensure that AF cells use less glucose, especially at the inner AF/NP interface, thereby allowing greater amount of glucose to bypass AF to diffuse into the NP to feed the cells in that disc region; this phenomenon has been previously reported in solid tumors which are anatomically analogous to disc tissue [14]. Third, our study has not established that lactate utilization as a biofuel is biologically essential. To demonstrate such is the case, AF-targeted genetic knockout of one of the lactate handling machineries, e.g. MCT-1 or LDH-B, in an in vivo model is required.

## Conclusions

Our new data suggest that lactate has been overlooked as an important biofuel in disc metabolism. Importantly, our findings also indicate the existence of lactate-dependent metabolic symbiosis between NP and AF of the IVD as a metabolic adaptation to efficiently recycle lactate. Such metabolic adaptation would not only prevent the accumulation of lactate as a waste product but also simultaneously generate the much-needed energy as well as precursors for biosynthesis to cells residing within the nutrient-poor disc environment. As a point of illustration, only two ATPs would be produced from glycolysis of each glucose molecule if lactate is eliminated as waste. This is miniscule compared to about 30 ATPs produced from one glucose if lactate is metabolized via OXPHOS.

One important implication of our model is that compromised or disrupted lactate symbiosis could contribute to metabolic disturbance and IDD. If disc lactate metabolic symbiosis is indeed determined to be vital for disc health, it will open a floodgate of new and important questions for future studies regarding disc metabolism, including identification of protein regulators and mechanistic pathways that control expression of key lactate handling molecular machinery and their enzymatic activities in lactate transport and metabolism. Verifying the importance of lactate metabolism by AF cells will guide the development of new therapeutic interventions to combat metabolic-related IDD disorders.

## Supplementary Information

**Additional file 1: Figure S1.** Lactate tolerance by AF cells in cell culture. Rabbit AF cells were grown on monolayer culture under physiologic nutrients, e.g. 1% FBS, 1.06mM glucose and varying exogenously added lactate concentrations (0-20 mM), for 48 hrs. Effects of different lactate concentrations on cell density and morphology (**A**) and cell viability as determined by CCK8 assay (**B**). Cell viability data are means SEM of three independent experiments (3 rabbits). **Figure S2.** Lactate import into HepG2 cells. ^14^C-lactate radioactive tracing to assess lactate import into HepG2 cell cultures grown in 1mM glucose and varying concentrations of ^14^C-lactate (1, 4mM) for one minute, washed with PBS, lysed and counted in scintillation fluid. **Figure S3.** Lactate conversion to pyruvate in AF cells in ex vivo disc organ. ^13^C-lactate tracing to pyruvate conversion in ex vivo rabbit disc organ culture containing 4 mM ^13^C-lactate in the culture media. Intracellular enrichment of ^13^C lactate or pyruvate AF cells from AF tissue of the ex vivo disc organ culture is reported as atomic percent excess (APE) of the total amount of lactate or pyruvate, e.g. 10% APE of pyruvate indicates 10% of total pyruvate contains ^13^C. Percent (%) APE shown. (M+1) indicates that one ^13^C carbon is present on lactate or pyruvate molecule. **Figure S4**. Lactate uptake and conversion to TCA intermediates and amino acids in rabbit, human, and rat AF cells. (**A**) ^13^C from rabbit AF cells cultured in 4 mM 3-^13^C-lactate and 1mM glucose was traced to amino acids glutamate, glutamine, and alanine. (**B**) Preferential lactate uptake and conversion to TCA intermediates by rabbit AF cells. ^13^C from ^13^C -Lactate or ^13^C -Glucose was traced by HRMS to TCA intermediates in rabbit AF cells grown in three different culture media. Note that ^13^C enrichments of succinate, fumarate, and malate from 1mM ^13^C -Glucose (black bars) were dramatically reduced in the presence of 4 mM unlabeled lactate (grey bars). Consistent with this result is that ^13^C enrichments of succinate, fumarate, and malate from 4mM ^13^C -Lactate were not affected in 1mM unlabeled glucose (white bars), indicating that AF cells preferentially utilize lactate when grown under physiologic nutrient condition of 1mM glucose and 4mM lactate. (**C**) ^13^C from human AF cells cultured in 4 mM 3-^13^C-lactate was traced to the TCA intermediate malate and the amino acid glutamate. (**D**) Caudal discs of Fischer 344 rats were injected with 3-^13^C-lactate (see method) and ^13^C was traced to lactate, malate, and glutamate in the AF tissues 3 days post injection. Intracellular enrichment of ^13^C on different metabolites in AF cells was measured in APE or atom percent excess. Data are means SEM of three independent experiments (three rats) for **D**, four experiments for **C** (four human disc specimen), and four experiments for **A**, **B** (four rabbits). **Figure S5.** Pharmacological profiling of OCR of rabbit AF cells in the absence and presence of lactate. OCR of AF cells (**A**) were measured by Seahorse XFe96 Extracellular Flux Analyzer at basal conditions and with serial administration of 1 M oligomycin, 0.3 M FCCP, 100 mM 2DG and 1 M rotenone. OCR was calculated and normalized to protein amount and the results were expressed as a mean of four different samples SEM. Individual parameters of OXPHOS (**B**) were derived from OCR profiles of AF cells lactate, as described in Materials and Methods. Addition of lactate did not significantly affect respiration reserved capacity (Res Cap), respiration total capacity (Tot Cap), non-glucose respiration (NG OCR) and non-mitochondrial oxygen consumption (NMR). Results are expressed as mean of four different samples (derived from four rabbits) SEM, * *p* < 0.05. **Figure S6.** Exogenously added lactate decreases matrix protein synthesis**.** Rabbit NP cell cultures exposed to physiological concentration of glucose (1mM) in the presence or absence of 4mM lactate. The presence of lactate decreases overall proteoglycan synthesis (^35^S-sulfate incorporation, *p*= 0.64, **A**), collagen matrix synthesis (^3^H-Lproline incorporation & collagenase sensitive fraction, *p*=0.21, **B**), and protein synthesis (^3^H-L-proline incorporation, *p*=0.1, **C**). **D**) Rabbit AF cells grown at 5% O_2_ in 5mM glucose, 5mM lactate, or 1mM glucose + 4mM lactate generated similar levels of total protein (black bars) and collagen (grey bars) synthesis. Results are expressed as mean of four different samples (derived from four rabbits) SEM. **Figure S7**. HK2 protein expression in AF and NP tissues. Western blot of hexokinase 2 (HK2) in rabbit AF and NP tissue protein extract (**A**) and their protein levels were quantified by normalizing to -actin as loading control (**B**). Data are means SD of three independent experiments (three rabbits). * p<0.05.

## Data Availability

All data generated or analyzed during this study are included in this published article [and its supplementary information files].

## Abbreviations

AF: Annulus fibrosus
FBS: Fetal bovine serum
HK: Hexokinase
IDD: Intervertebral disc degeneration
IVD: Intervertebral disc
LDH: Lactate dehydrogenase
MCT: Monocarboxylate transporter
NP: Nucleus pulposus
OXPHOS: Oxidative phosphorylation
PDH: Pyruvate dehydrogenase
PDK: Pyruvate dehydrogenase kinase
PG: Proteoglycan
TCA: Tricarboxylic acid
