# Supplementary figures and images for: Lactate oxidative phosphorylation by annulus fibrosus cells: evidence for lactate-dependent metabolic symbiosis in intervertebral discs
Source: Arthritis Res Ther. 2021 May 21;23:145. doi: 10.1186/s13075-021-02501-2 (PMC8139157; doi:10.1186/s13075-021-02501-2)

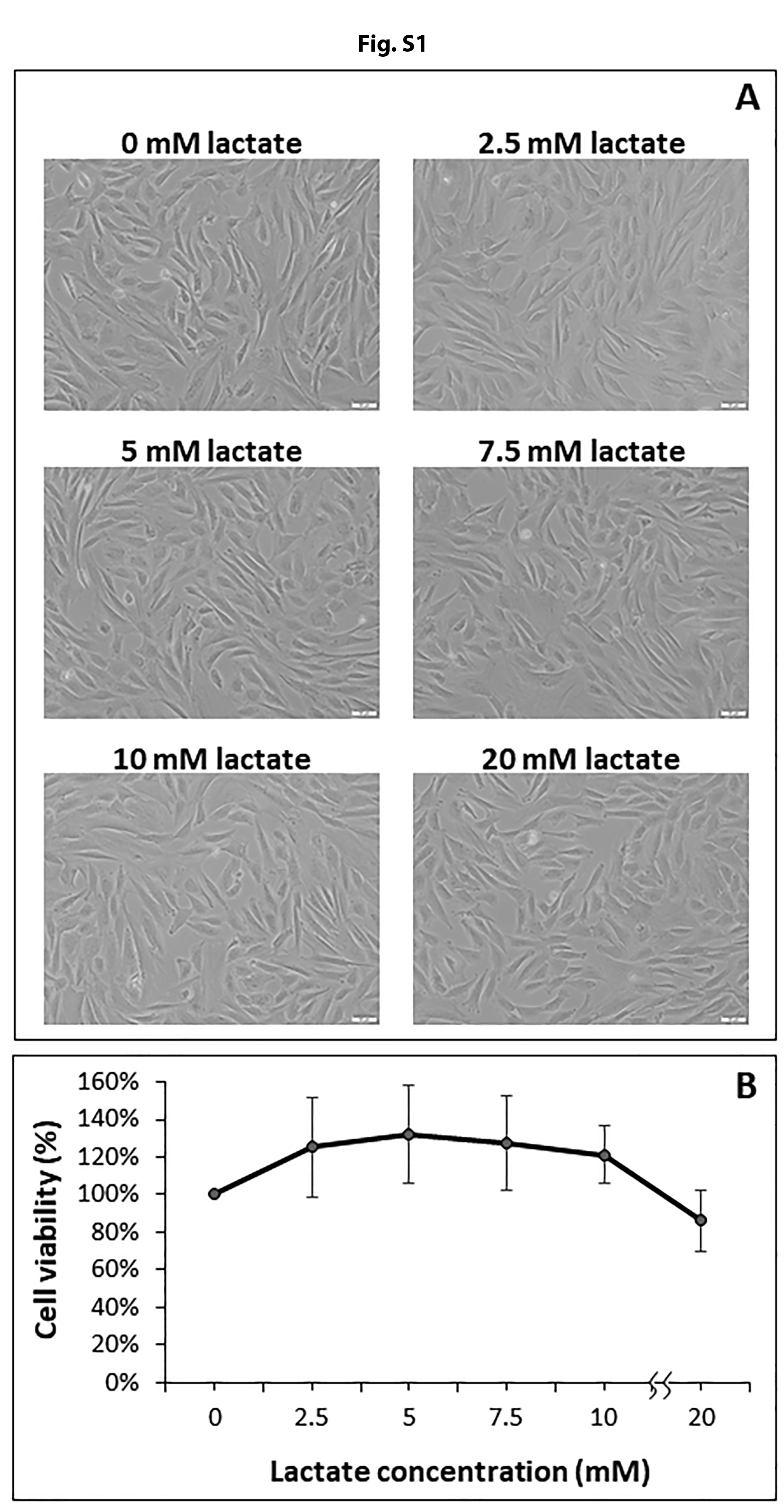

Supplement: Supplementary file 1 — Additional file 1: Figure S1. Lactate tolerance by AF cells in cell culture. Rabbit AF cells were grown on monolayer culture under physiologic nutrients, e.g. 1% FBS, 1.06mM glucose and varying exogenously added lactate concentrations (0-20 mM), for 48 hrs. Effects of different lactate concentrations on cell density and morphology (A) and cell viability as determined by CCK8 assay (B). Cell viability data are means SEM of three independent experiments (3 rabbits). Figure S2. Lactate import into HepG2 cells. 14C-lactate radioactive tracing to assess lactate import into HepG2 cell cultures grown in 1mM glucose and varying concentrations of 14C-lactate (1, 4mM) for one minute, washed with PBS, lysed and counted in scintillation fluid. Figure S3. Lactate conversion to pyruvate in AF cells in ex vivo disc organ. 13C-lactate tracing to pyruvate conversion in ex vivo rabbit disc organ culture containing 4 mM 13C-lactate in the culture media. Intracellular enrichment of 13C lactate or pyruvate AF cells from AF tissue of the ex vivo disc organ culture is reported as atomic percent excess (APE) of the total amount of lactate or pyruvate, e.g. 10% APE of pyruvate indicates 10% of total pyruvate contains 13C. Percent (%) APE shown. (M+1) indicates that one 13C carbon is present on lactate or pyruvate molecule. Figure S4. Lactate uptake and conversion to TCA intermediates and amino acids in rabbit, human, and rat AF cells. (A) 13C from rabbit AF cells cultured in 4 mM 3-13C-lactate and 1mM glucose was traced to amino acids glutamate, glutamine, and alanine. (B) Preferential lactate uptake and conversion to TCA intermediates by rabbit AF cells. 13C from 13C -Lactate or 13C -Glucose was traced by HRMS to TCA intermediates in rabbit AF cells grown in three different culture media. Note that 13C enrichments of succinate, fumarate, and malate from 1mM 13C -Glucose (black bars) were dramatically reduced in the presence of 4 mM unlabeled lactate (grey bars). Consistent with this result [file 13075_2021_2501_MOESM1_ESM.zip › XFig S1-cell viability_ESM.tif]

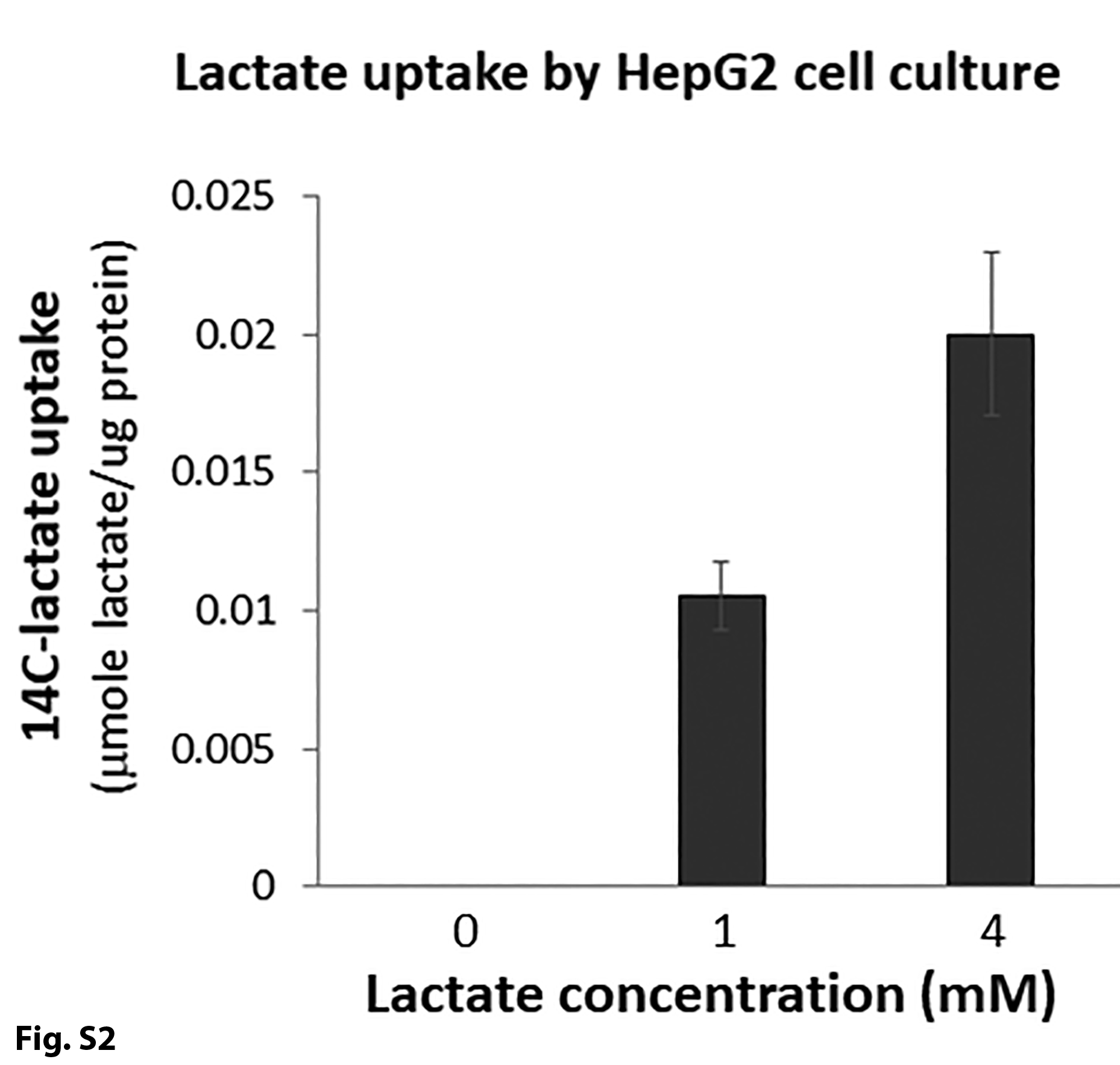

Supplement: Supplementary file 1 — Additional file 1: Figure S1. Lactate tolerance by AF cells in cell culture. Rabbit AF cells were grown on monolayer culture under physiologic nutrients, e.g. 1% FBS, 1.06mM glucose and varying exogenously added lactate concentrations (0-20 mM), for 48 hrs. Effects of different lactate concentrations on cell density and morphology (A) and cell viability as determined by CCK8 assay (B). Cell viability data are means SEM of three independent experiments (3 rabbits). Figure S2. Lactate import into HepG2 cells. 14C-lactate radioactive tracing to assess lactate import into HepG2 cell cultures grown in 1mM glucose and varying concentrations of 14C-lactate (1, 4mM) for one minute, washed with PBS, lysed and counted in scintillation fluid. Figure S3. Lactate conversion to pyruvate in AF cells in ex vivo disc organ. 13C-lactate tracing to pyruvate conversion in ex vivo rabbit disc organ culture containing 4 mM 13C-lactate in the culture media. Intracellular enrichment of 13C lactate or pyruvate AF cells from AF tissue of the ex vivo disc organ culture is reported as atomic percent excess (APE) of the total amount of lactate or pyruvate, e.g. 10% APE of pyruvate indicates 10% of total pyruvate contains 13C. Percent (%) APE shown. (M+1) indicates that one 13C carbon is present on lactate or pyruvate molecule. Figure S4. Lactate uptake and conversion to TCA intermediates and amino acids in rabbit, human, and rat AF cells. (A) 13C from rabbit AF cells cultured in 4 mM 3-13C-lactate and 1mM glucose was traced to amino acids glutamate, glutamine, and alanine. (B) Preferential lactate uptake and conversion to TCA intermediates by rabbit AF cells. 13C from 13C -Lactate or 13C -Glucose was traced by HRMS to TCA intermediates in rabbit AF cells grown in three different culture media. Note that 13C enrichments of succinate, fumarate, and malate from 1mM 13C -Glucose (black bars) were dramatically reduced in the presence of 4 mM unlabeled lactate (grey bars). Consistent with this result [file 13075_2021_2501_MOESM1_ESM.zip › XFig S2- HepG2 cells_ESM.tif]

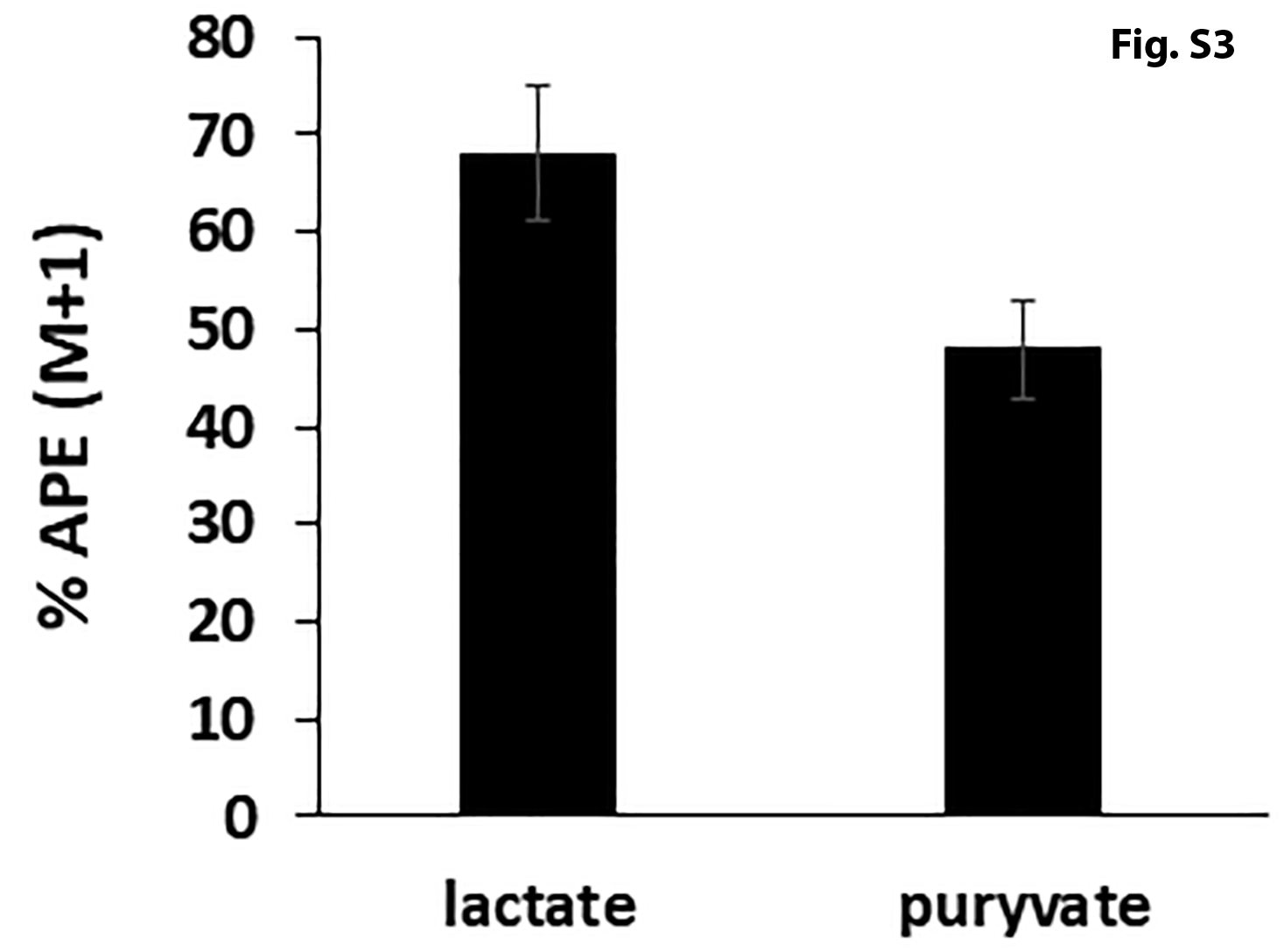

Supplement: Supplementary file 1 — Additional file 1: Figure S1. Lactate tolerance by AF cells in cell culture. Rabbit AF cells were grown on monolayer culture under physiologic nutrients, e.g. 1% FBS, 1.06mM glucose and varying exogenously added lactate concentrations (0-20 mM), for 48 hrs. Effects of different lactate concentrations on cell density and morphology (A) and cell viability as determined by CCK8 assay (B). Cell viability data are means SEM of three independent experiments (3 rabbits). Figure S2. Lactate import into HepG2 cells. 14C-lactate radioactive tracing to assess lactate import into HepG2 cell cultures grown in 1mM glucose and varying concentrations of 14C-lactate (1, 4mM) for one minute, washed with PBS, lysed and counted in scintillation fluid. Figure S3. Lactate conversion to pyruvate in AF cells in ex vivo disc organ. 13C-lactate tracing to pyruvate conversion in ex vivo rabbit disc organ culture containing 4 mM 13C-lactate in the culture media. Intracellular enrichment of 13C lactate or pyruvate AF cells from AF tissue of the ex vivo disc organ culture is reported as atomic percent excess (APE) of the total amount of lactate or pyruvate, e.g. 10% APE of pyruvate indicates 10% of total pyruvate contains 13C. Percent (%) APE shown. (M+1) indicates that one 13C carbon is present on lactate or pyruvate molecule. Figure S4. Lactate uptake and conversion to TCA intermediates and amino acids in rabbit, human, and rat AF cells. (A) 13C from rabbit AF cells cultured in 4 mM 3-13C-lactate and 1mM glucose was traced to amino acids glutamate, glutamine, and alanine. (B) Preferential lactate uptake and conversion to TCA intermediates by rabbit AF cells. 13C from 13C -Lactate or 13C -Glucose was traced by HRMS to TCA intermediates in rabbit AF cells grown in three different culture media. Note that 13C enrichments of succinate, fumarate, and malate from 1mM 13C -Glucose (black bars) were dramatically reduced in the presence of 4 mM unlabeled lactate (grey bars). Consistent with this result [file 13075_2021_2501_MOESM1_ESM.zip › XFig S3- 13C-lactate pyruvate FSU soaked_ESM.tif]

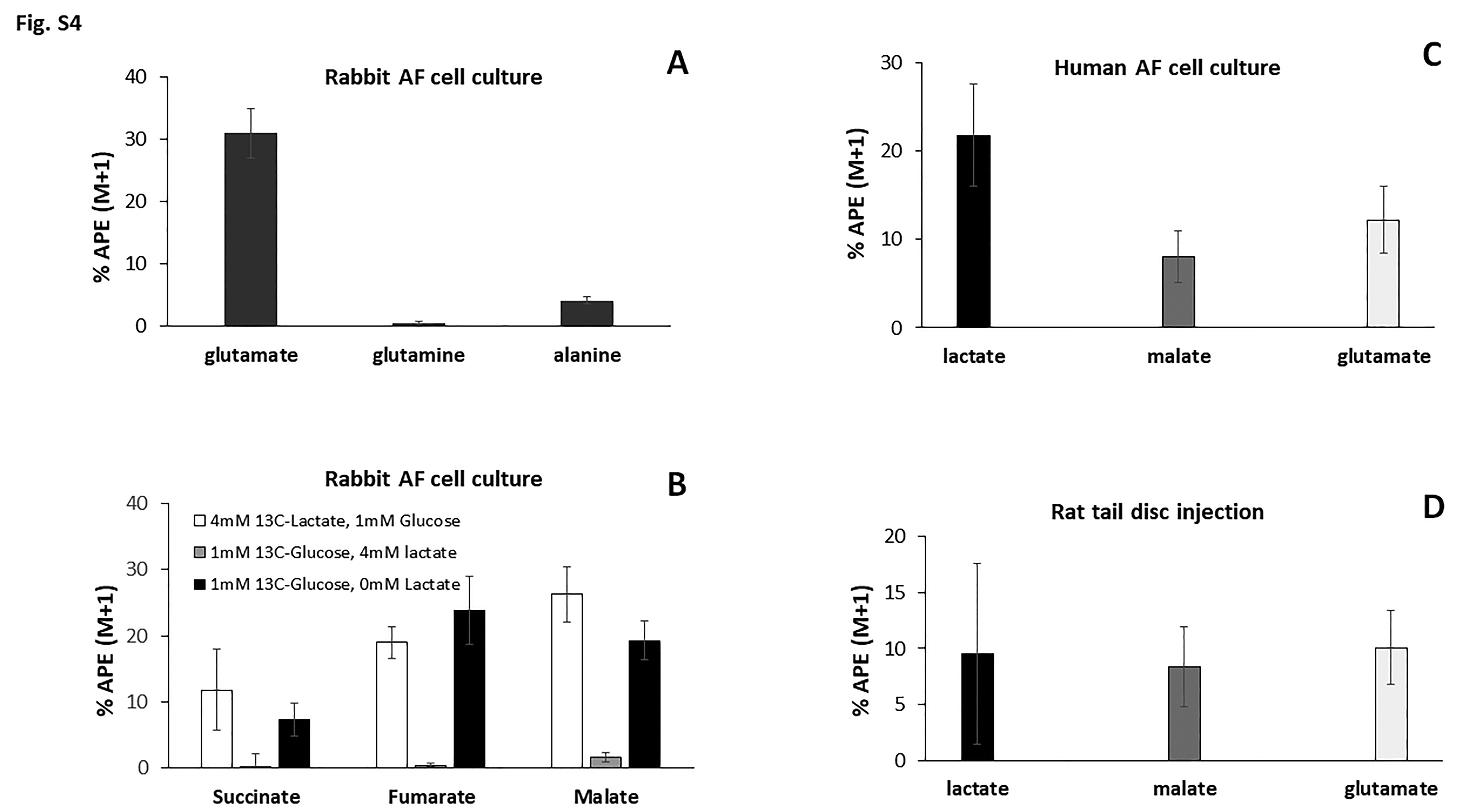

Supplement: Supplementary file 1 — Additional file 1: Figure S1. Lactate tolerance by AF cells in cell culture. Rabbit AF cells were grown on monolayer culture under physiologic nutrients, e.g. 1% FBS, 1.06mM glucose and varying exogenously added lactate concentrations (0-20 mM), for 48 hrs. Effects of different lactate concentrations on cell density and morphology (A) and cell viability as determined by CCK8 assay (B). Cell viability data are means SEM of three independent experiments (3 rabbits). Figure S2. Lactate import into HepG2 cells. 14C-lactate radioactive tracing to assess lactate import into HepG2 cell cultures grown in 1mM glucose and varying concentrations of 14C-lactate (1, 4mM) for one minute, washed with PBS, lysed and counted in scintillation fluid. Figure S3. Lactate conversion to pyruvate in AF cells in ex vivo disc organ. 13C-lactate tracing to pyruvate conversion in ex vivo rabbit disc organ culture containing 4 mM 13C-lactate in the culture media. Intracellular enrichment of 13C lactate or pyruvate AF cells from AF tissue of the ex vivo disc organ culture is reported as atomic percent excess (APE) of the total amount of lactate or pyruvate, e.g. 10% APE of pyruvate indicates 10% of total pyruvate contains 13C. Percent (%) APE shown. (M+1) indicates that one 13C carbon is present on lactate or pyruvate molecule. Figure S4. Lactate uptake and conversion to TCA intermediates and amino acids in rabbit, human, and rat AF cells. (A) 13C from rabbit AF cells cultured in 4 mM 3-13C-lactate and 1mM glucose was traced to amino acids glutamate, glutamine, and alanine. (B) Preferential lactate uptake and conversion to TCA intermediates by rabbit AF cells. 13C from 13C -Lactate or 13C -Glucose was traced by HRMS to TCA intermediates in rabbit AF cells grown in three different culture media. Note that 13C enrichments of succinate, fumarate, and malate from 1mM 13C -Glucose (black bars) were dramatically reduced in the presence of 4 mM unlabeled lactate (grey bars). Consistent with this result [file 13075_2021_2501_MOESM1_ESM.zip › XFig S4-human rabbit rat 13Clactate tracing_ESM.tif]

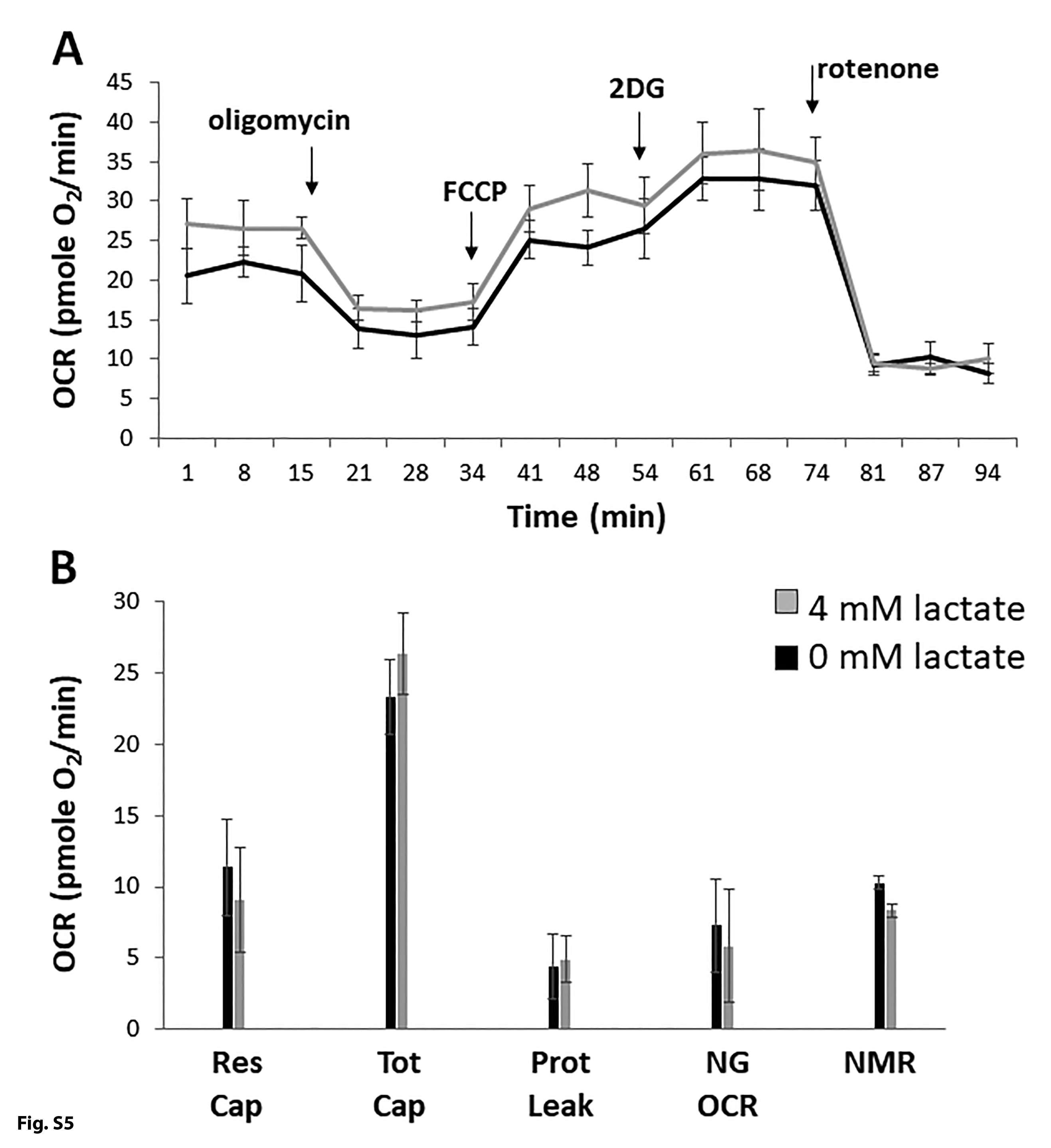

Supplement: Supplementary file 1 — Additional file 1: Figure S1. Lactate tolerance by AF cells in cell culture. Rabbit AF cells were grown on monolayer culture under physiologic nutrients, e.g. 1% FBS, 1.06mM glucose and varying exogenously added lactate concentrations (0-20 mM), for 48 hrs. Effects of different lactate concentrations on cell density and morphology (A) and cell viability as determined by CCK8 assay (B). Cell viability data are means SEM of three independent experiments (3 rabbits). Figure S2. Lactate import into HepG2 cells. 14C-lactate radioactive tracing to assess lactate import into HepG2 cell cultures grown in 1mM glucose and varying concentrations of 14C-lactate (1, 4mM) for one minute, washed with PBS, lysed and counted in scintillation fluid. Figure S3. Lactate conversion to pyruvate in AF cells in ex vivo disc organ. 13C-lactate tracing to pyruvate conversion in ex vivo rabbit disc organ culture containing 4 mM 13C-lactate in the culture media. Intracellular enrichment of 13C lactate or pyruvate AF cells from AF tissue of the ex vivo disc organ culture is reported as atomic percent excess (APE) of the total amount of lactate or pyruvate, e.g. 10% APE of pyruvate indicates 10% of total pyruvate contains 13C. Percent (%) APE shown. (M+1) indicates that one 13C carbon is present on lactate or pyruvate molecule. Figure S4. Lactate uptake and conversion to TCA intermediates and amino acids in rabbit, human, and rat AF cells. (A) 13C from rabbit AF cells cultured in 4 mM 3-13C-lactate and 1mM glucose was traced to amino acids glutamate, glutamine, and alanine. (B) Preferential lactate uptake and conversion to TCA intermediates by rabbit AF cells. 13C from 13C -Lactate or 13C -Glucose was traced by HRMS to TCA intermediates in rabbit AF cells grown in three different culture media. Note that 13C enrichments of succinate, fumarate, and malate from 1mM 13C -Glucose (black bars) were dramatically reduced in the presence of 4 mM unlabeled lactate (grey bars). Consistent with this result [file 13075_2021_2501_MOESM1_ESM.zip › XFig S5- Seahorse_ESM.tif]

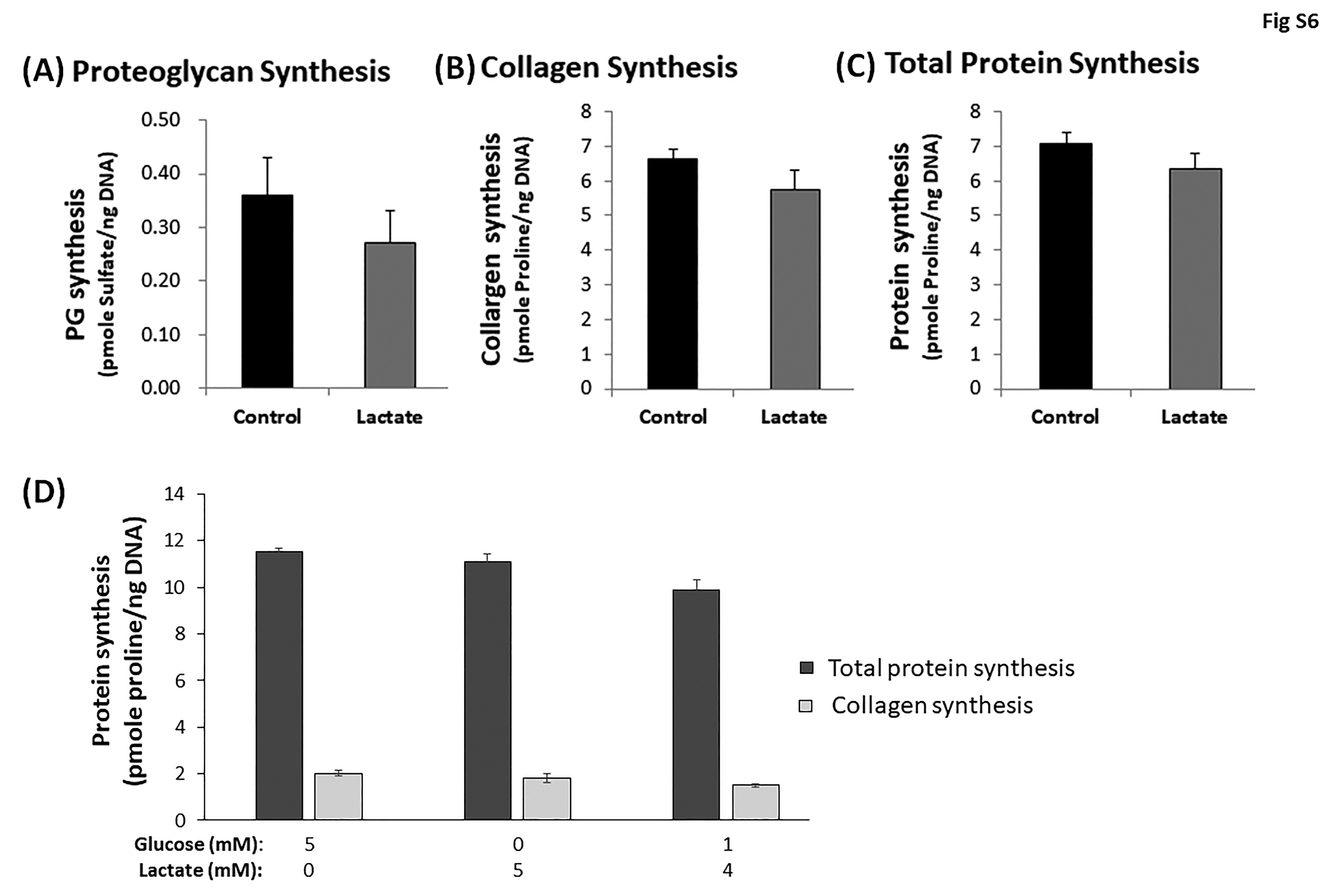

Supplement: Supplementary file 1 — Additional file 1: Figure S1. Lactate tolerance by AF cells in cell culture. Rabbit AF cells were grown on monolayer culture under physiologic nutrients, e.g. 1% FBS, 1.06mM glucose and varying exogenously added lactate concentrations (0-20 mM), for 48 hrs. Effects of different lactate concentrations on cell density and morphology (A) and cell viability as determined by CCK8 assay (B). Cell viability data are means SEM of three independent experiments (3 rabbits). Figure S2. Lactate import into HepG2 cells. 14C-lactate radioactive tracing to assess lactate import into HepG2 cell cultures grown in 1mM glucose and varying concentrations of 14C-lactate (1, 4mM) for one minute, washed with PBS, lysed and counted in scintillation fluid. Figure S3. Lactate conversion to pyruvate in AF cells in ex vivo disc organ. 13C-lactate tracing to pyruvate conversion in ex vivo rabbit disc organ culture containing 4 mM 13C-lactate in the culture media. Intracellular enrichment of 13C lactate or pyruvate AF cells from AF tissue of the ex vivo disc organ culture is reported as atomic percent excess (APE) of the total amount of lactate or pyruvate, e.g. 10% APE of pyruvate indicates 10% of total pyruvate contains 13C. Percent (%) APE shown. (M+1) indicates that one 13C carbon is present on lactate or pyruvate molecule. Figure S4. Lactate uptake and conversion to TCA intermediates and amino acids in rabbit, human, and rat AF cells. (A) 13C from rabbit AF cells cultured in 4 mM 3-13C-lactate and 1mM glucose was traced to amino acids glutamate, glutamine, and alanine. (B) Preferential lactate uptake and conversion to TCA intermediates by rabbit AF cells. 13C from 13C -Lactate or 13C -Glucose was traced by HRMS to TCA intermediates in rabbit AF cells grown in three different culture media. Note that 13C enrichments of succinate, fumarate, and malate from 1mM 13C -Glucose (black bars) were dramatically reduced in the presence of 4 mM unlabeled lactate (grey bars). Consistent with this result [file 13075_2021_2501_MOESM1_ESM.zip › XFig S6- NP matrix synthesis_ESM.tif]

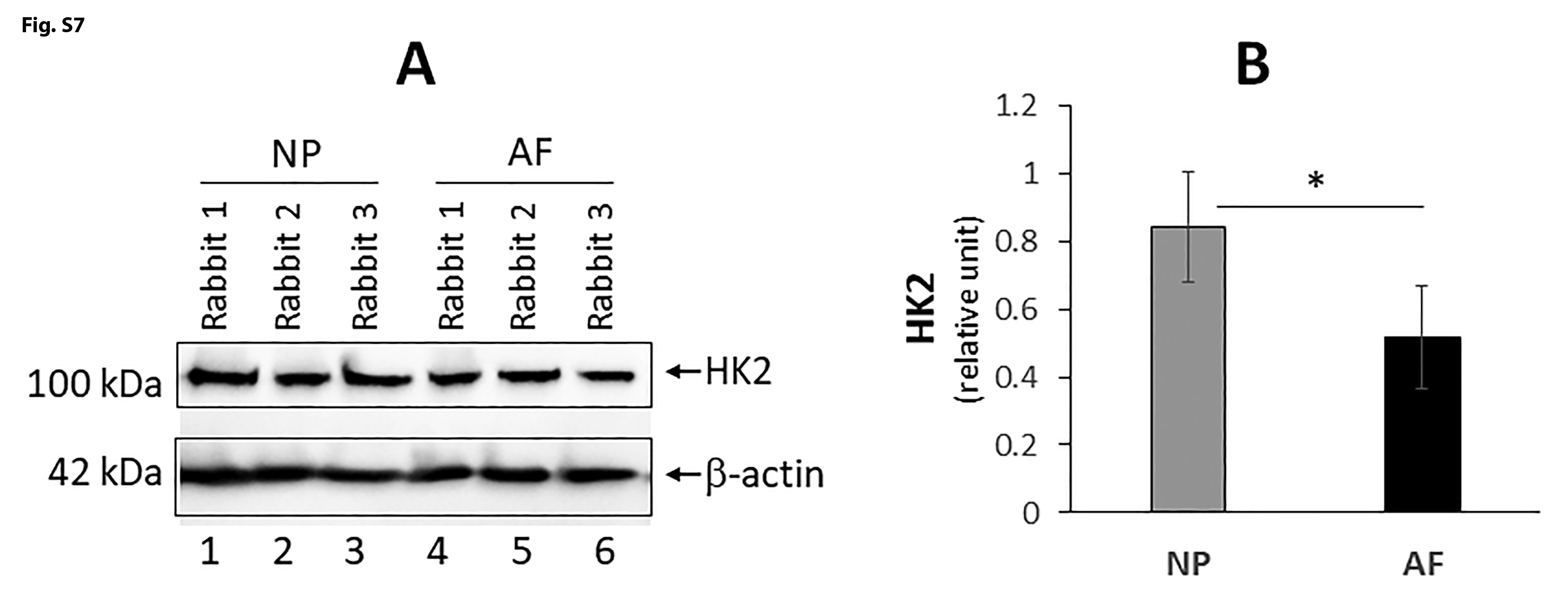

Supplement: Supplementary file 1 — Additional file 1: Figure S1. Lactate tolerance by AF cells in cell culture. Rabbit AF cells were grown on monolayer culture under physiologic nutrients, e.g. 1% FBS, 1.06mM glucose and varying exogenously added lactate concentrations (0-20 mM), for 48 hrs. Effects of different lactate concentrations on cell density and morphology (A) and cell viability as determined by CCK8 assay (B). Cell viability data are means SEM of three independent experiments (3 rabbits). Figure S2. Lactate import into HepG2 cells. 14C-lactate radioactive tracing to assess lactate import into HepG2 cell cultures grown in 1mM glucose and varying concentrations of 14C-lactate (1, 4mM) for one minute, washed with PBS, lysed and counted in scintillation fluid. Figure S3. Lactate conversion to pyruvate in AF cells in ex vivo disc organ. 13C-lactate tracing to pyruvate conversion in ex vivo rabbit disc organ culture containing 4 mM 13C-lactate in the culture media. Intracellular enrichment of 13C lactate or pyruvate AF cells from AF tissue of the ex vivo disc organ culture is reported as atomic percent excess (APE) of the total amount of lactate or pyruvate, e.g. 10% APE of pyruvate indicates 10% of total pyruvate contains 13C. Percent (%) APE shown. (M+1) indicates that one 13C carbon is present on lactate or pyruvate molecule. Figure S4. Lactate uptake and conversion to TCA intermediates and amino acids in rabbit, human, and rat AF cells. (A) 13C from rabbit AF cells cultured in 4 mM 3-13C-lactate and 1mM glucose was traced to amino acids glutamate, glutamine, and alanine. (B) Preferential lactate uptake and conversion to TCA intermediates by rabbit AF cells. 13C from 13C -Lactate or 13C -Glucose was traced by HRMS to TCA intermediates in rabbit AF cells grown in three different culture media. Note that 13C enrichments of succinate, fumarate, and malate from 1mM 13C -Glucose (black bars) were dramatically reduced in the presence of 4 mM unlabeled lactate (grey bars). Consistent with this result [file 13075_2021_2501_MOESM1_ESM.zip › XFig S7- HK2 western_ESM.tif]
